# Acute caffeine supplementation in rugby players: a systematic review and meta-analysis of physical performance, sport-specific performance, perceptual responses, and physiological markers

**DOI:** 10.3389/fnut.2026.1884686

**Published:** 2026-07-03

**Authors:** Jun Li, Dawei Zhou, Tianle Wang, Zeyong Liu, Zhuo Zeng, Caizheng Yue, Cong Huang, Weixin Chen, Junyi Shu, Fanhao Meng

**Affiliations:** 1 School of Physical Education, Shaanxi Normal University, Xi’an, China; 2 Department of Physical Education, Lanzhou University, Lanzhou, China; 3 School of Art, Beijing Sport University, Beijing, China; 4 School of Strength and Conditioning, Beijing Sport University, Beijing, China; 5 Department of Sport and Sport Science, University of Freiburg, Freiburg, Germany

**Keywords:** caffeine, ergogenic aids, meta-analysis, perceived exertion, rugby, sports nutrition, sprint performance

## Abstract

**Background:**

Rugby is a collision-based team sport requiring repeated high-intensity sprinting, technical execution, and physical contact under fatigue. Although caffeine is used as an ergogenic supplement, evidence from mixed team-sport populations may not be applicable to rugby. This systematic review evaluated the effects of acute caffeine supplementation on physical and sport-specific performance, perceptual responses, and physiological markers in rugby players.

**Methods:**

PubMed, Web of Science, Cochrane Library, Scopus, and Embase were searched from inception to May 1, 2026. Randomized placebo-controlled studies examining acute caffeine supplementation in rugby players were included in the quantitative synthesis, whereas observational studies related to sleep and recovery were narratively summarized. Standardized mean differences were calculated as Hedges’ g with 95% confidence intervals. Three-level random-effects models accounted for dependent effect sizes. Methodological quality and risk of bias in randomized trials were assessed using the PEDro scale and RoB 2, respectively. Observational studies were assessed using the JBI checklist, and certainty of evidence was evaluated using GRADE.

**Results:**

Fifteen studies were included, and 13 randomized placebo-controlled trials contributed data to the meta-analysis. Acute caffeine supplementation was associated with small improvements in sprint performance (*k* = 11, Hedges’ *g* = −0.41, 95% CI: −0.73 to −0.08, *p* = 0.02) and lower rating of perceived exertion (RPE) measured during or immediately after rugby-specific or resistance-exercise protocols (*k* = 9, Hedges’ *g* = −0.40, 95% CI: −0.73 to −0.06, *p* = 0.02). A large positive effect was observed for passing accuracy (*k* = 9, Hedges’ *g* = 1.65, 95% CI: 0.69 to 2.61, *p* = 0.004), but this finding showed high heterogeneity and potential small-study effects. No significant effects were found for jumping performance, strength performance, metabolic markers, or hormonal markers. Subgroup analyses did not identify moderating effects of rugby code, dose, form of administration, or competitive level. Observational evidence suggested possible sleep and recovery concerns following caffeine exposure.

**Conclusion:**

In rugby players, acute caffeine may improve sprint performance and passing accuracy while reducing RPE measured during or immediately after rugby-specific or resistance-exercise protocols. However, certainty of evidence ranged from low to very low, and findings should be interpreted cautiously.

**Systematic review registration:**

https://www.crd.york.ac.uk/prospero/, PROSPERO: CRD420261371265.

## Graphical abstract

**Figure:**
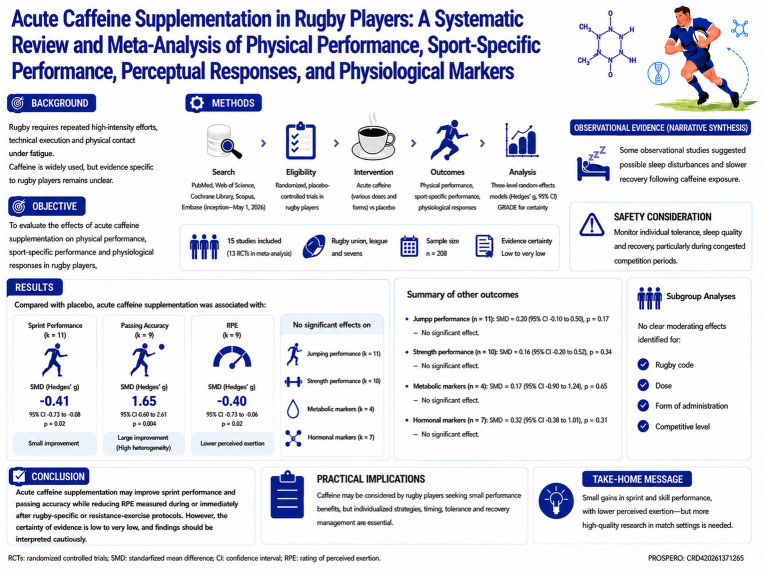
Graphical abstract

## 1 Introduction

Rugby is an intermittent collision sport with several competitive codes, including rugby sevens, rugby union, and rugby league (1). Although these codes differ in match duration, number of players, tactical structure, and physical demands, they commonly require repeated high-intensity running, sprinting, rapid changes of direction, technical execution, and frequent physical contact under fatigue (2, 3). Rugby sevens is generally characterized by a faster match tempo and greater relative running and sprinting demands, whereas rugby union and rugby league involve longer match durations and more sustained exposure to collisions, contact events, and more position-specific demands (4–6). Therefore, rugby players must not only generate repeated high-intensity efforts but also maintain technical accuracy and decision-making under fatigue and physical contact.

Caffeine is one of the most widely used ergogenic supplements in sport (7–9). Its ergogenic effects may involve central and peripheral mechanisms, including adenosine receptor antagonism, reduced perceived fatigue, enhanced motor unit recruitment, improved neuromuscular activation, and increased calcium release from the sarcoplasmic reticulum, thereby supporting force production and high-intensity exercise performance (9). Previous systematic reviews and meta-analyses have suggested that acute caffeine supplementation may improve repeated sprint ability (10), jumping performance (11), muscular strength (12), and some team-sport-related outcomes (13). However, rugby combines intermittent high-intensity running with frequent collisions, contact-induced fatigue, and sport-specific technical demands, which makes it distinct from many non-collision team sports (14). Compared with non-collision team sports, the ergogenic effects of caffeine in rugby may be influenced by additional factors such as contact load, match format, playing position, testing context, and the need to maintain technical execution under defensive pressure. Therefore, findings from endurance exercise, resistance exercise, or mixed team-sport populations may not be directly generalizable to rugby.

Several randomized controlled studies have investigated the effects of acute caffeine supplementation in rugby players across physical performance, sport-specific performance, perceptual responses, and physiological markers, including sprint performance, repeated high-intensity efforts, passing accuracy, reactive agility, RPE measured during or immediately after rugby-specific or resistance-exercise protocols, and metabolic and hormonal markers (15–18). However, findings remain inconsistent across studies, especially for general physical performance outcomes such as jumping, strength, and power, as well as for perceptual responses, physiological markers, and sport-specific technical outcomes. These discrepancies may be partly explained by differences in rugby code, competitive level, caffeine dose, mode of administration, timing of administration, and outcome selection (15–17). In addition, some studies used standardized laboratory or simulated rugby protocols, whereas others examined match-related or field-based outcomes, which may influence both the interpretation and practical relevance of the findings.

Previous studies on caffeine and team-sport performance have generally included rugby players within broader mixed-sport samples, together with athletes from sports such as soccer, basketball, volleyball, and handball (19, 20). Although these studies provide useful general evidence, this approach may obscure the specific physiological and technical demands of rugby as a collision sport. While repeated sprinting and technical execution under fatigue are also important in other team sports, the frequent collision and contact demands of rugby may create a distinct performance context in which the effects of caffeine require specific evaluation (2, 3). Therefore, a rugby-specific synthesis is needed to clarify whether acute caffeine supplementation provides meaningful benefits for rugby players and to determine which outcomes are most likely to be affected. By focusing specifically on rugby players, the present review provides rugby-specific pooled estimates for outcomes directly relevant to rugby and uses a three-level meta-analytic model to account for dependent effect sizes within individual studies.

This systematic review and meta-analysis evaluated the effects of acute caffeine supplementation on physical performance, sport-specific performance, perceptual responses, and physiological markers in rugby players. When sufficient data were available, exploratory subgroup analyses examined whether these effects differed by rugby code, caffeine dose, form of administration, and competitive level.

## 2 Methods

### 2.1 Search strategy

This systematic review and meta-analysis was conducted in accordance with the PRISMA 2020 guidelines, and the completed checklist is provided in Supplementary Table 1 (21). The study protocol was registered in PROSPERO under the registration number CRD420261371265. Two researchers (LJ and ZDW) independently conducted the literature search and cross-checked the results. The following databases were searched: PubMed, Web of Science Core Collection, Cochrane Library, Scopus, and Embase. The search period covered all records from database inception to May 1, 2026. No restrictions on publication region or language were applied during the search stage; however, only original studies with accessible full texts in English were included during full-text screening. The core search strategy was as follows: (rugby OR “rugby union” OR “rugby league” OR “rugby sevens”) AND (caffeine OR caffeinated OR coffee OR “caffeinated beverage” OR “energy drink” OR “caffeine gum” OR “caffeine mouth rinse”). The detailed search strategies are presented in Supplementary Table 2.

### 2.2 Inclusion and exclusion criteria

The inclusion and exclusion criteria were developed according to the PICOS framework (22). Studies were included if they met the following criteria: (1) the participants were rugby players, including rugby union, rugby league, or rugby sevens players; (2) the intervention involved a single dose of acute caffeine supplementation before testing or exercise; (3) the control condition was a placebo or a matched caffeine-free vehicle containing the same non-caffeine ingredients; (4) the outcomes included physical performance, sport-specific performance, perceptual responses, or physiological markers; (5) the primary meta-analysis included randomized, placebo-controlled studies of acute caffeine supplementation and observational studies were used only for the narrative synthesis of sleep- and recovery-related outcomes and were not included in the main effect-size pooling or the GRADE assessment of the primary outcomes.

Studies were excluded if they met any of the following criteria: (1) the population, intervention, or control condition did not meet the PICOS criteria; (2) the study did not report outcomes of interest or did not provide sufficient data for effect-size calculation; or (3) the article was a review, meta-analysis, conference abstract, commentary, case report, animal study, or other non-original study.

### 2.3 Study selection and data extraction

After duplicates were removed, two researchers (LJ and ZDW) independently screened the titles, abstracts, and full texts. Any disagreements were first resolved through discussion and, when necessary, adjudicated by a third researcher (HC). The final study selection process was presented using a PRISMA flow diagram (21).

Two researchers (LJ and ZDW) independently extracted study characteristics and outcome data using a predesigned data extraction form. The extracted information included first author, publication year, country or region, rugby code, study design, participant characteristics such as sample size, sex, age, and competitive level, caffeine dose, form of supplementation, timing of administration, washout period, and primary outcome measures.

For continuous outcomes, the sample size, mean, and standard deviation under the caffeine and control conditions were extracted. When means and standard deviations were not directly reported, they were converted from standard errors, 95% confidence intervals, *p* values, or other available statistics (23). If the study authors did not respond to data requests and graphical information was available, data were extracted from figures using WebPlotDigitizer 4.1. The extracted graphical data were cross-checked by two researchers (LJ and ZDW).

### 2.4 Outcomes and data processing

Outcomes were classified into four categories: physical performance, sport-specific performance, perceptual responses, and physiological markers. When a study reported multiple similar measures within the same outcome construct, the prespecified representative measure was preferentially extracted. When different outcome categories were reported, they were extracted separately, and the dependence among multiple effect sizes from the same study was accounted for in the statistical analysis. For studies with multiple assessment time points, the primary time point prespecified in the original article was extracted. If this was not specified, data measured immediately after completion of the exercise test or at the final assessment were used.

For sprint performance, single sprint time, total repeated sprint time, high-speed sprint distance, or fatigue index was extracted (15, 24–26). For jumping performance, countermovement jump, squat jump, drop jump height, or jump power was extracted (15, 17, 18). For strength performance, isometric mid-thigh pull peak force, or the number of repetitions to failure in the squat or bench press, was extracted (17, 18). For sport-specific technical performance, passing accuracy or the total score of a motor skills test was extracted (25–28). RPE was classified and analyzed separately as a perceptual response. The pooled analysis included RPE values obtained during or after rugby-specific exercise protocols and resistance-exercise repetitions-to-failure tests. When multiple measurements were reported, the final or post-exercise value was extracted unless another primary assessment was prespecified (17, 18, 25, 28–30). For physiological responses, blood lactate, blood glucose, and free fatty acids were extracted as metabolic outcomes; whereas plasma epinephrine, salivary testosterone, and salivary cortisol were classified as hormonal markers (15, 24). For time-to-completion outcomes, a negative effect size indicated improved performance. For outcomes such as jumping performance, strength performance, and passing accuracy, a positive effect size indicated improved performance.

### 2.5 Risk of bias and certainty of evidence assessment

Two researchers (LJ and ZDW) independently assessed the methodological quality, risk of bias, and certainty of evidence of the included studies. Any disagreements were resolved through discussion and, when necessary, adjudicated by a third researcher (WTL).

The methodological quality of randomized controlled trials was assessed using the PEDro scale (31). The scale consists of 11 items, of which item 1 assesses external validity and is not included in the total score. The remaining 10 items assess internal validity and the adequacy of statistical reporting, yielding a maximum score of 10. Higher scores indicate better methodological quality. Risk of bias was assessed using the Cochrane RoB 2 tool. The evaluated domains included the randomization process, deviations from intended interventions, missing outcome data, outcome measurement, and selective reporting. The overall risk of bias was judged as “low risk,” “some concerns,” or “high risk” (32). Sleep-related observational studies were used only for supplementary narrative description. These studies were assessed using the JBI Critical Appraisal Checklist for Analytical Cross-Sectional Studies and were not included in the PEDro assessment, RoB 2 assessment, or GRADE evaluation of the primary outcomes (33).

The certainty of evidence for the primary outcomes was assessed using the GRADE approach and was downgraded according to risk of bias, inconsistency, indirectness, imprecision, and publication bias (34).

### 2.6 Statistical analysis

All statistical analyses were performed using R software, with the metafor package used mainly for effect-size calculation and meta-analysis (35). The included intervention studies employed randomized crossover designs, which generate paired data. For multi-arm studies, the caffeine-containing condition was compared with the matched caffeine-free vehicle. For each outcome, the standardized mean difference (SMD) was calculated as the mean difference between the caffeine and control conditions divided by the pooled standard deviation of the two conditions. To account for the paired nature of the data, the variance of the SMD was calculated using a formula that incorporates the within-subject correlation (*r*). This method adjusts the variance for the dependence between measurements from the same participant (36, 37). When studies did not report the necessary within-subject correlation coefficient, a conservative estimate of *r* = 0.5 was used for the primary analysis (36, 37).

Continuous outcomes were expressed as standardized mean differences with 95% confidence intervals, and Hedges’ *g* was used to correct for small-sample bias (38). The magnitude of effect sizes was interpreted as follows: |*g*| < 0.20 indicated a trivial effect, 0.20 ≤ |*g*| < 0.50 a small effect, 0.50 ≤ |*g*| < 0.80 a moderate effect, and |g| ≥ 0.80 a large effect (39). Statistical power was also calculated for the main effects and pooled subgroup effects to help determine whether non-significant findings might be attributable to insufficient sample size (40).

Because individual studies could report multiple outcomes, time points, or dose conditions, a three-level random-effects model was used to account for dependence among effect sizes (41). Level 1 represented sampling error, Level 2 represented within-study variation among multiple effect sizes from the same study, and Level 3 represented between-study variation (42, 43). Model parameters were estimated using restricted maximum likelihood (35). Heterogeneity was assessed using variance components and total *I*^2^, with values of 0–30%, 30–50%, and >50% interpreted as low, moderate, and high heterogeneity, respectively (44).

Preplanned subgroup analyses were conducted according to rugby code, caffeine dose, form of supplementation, and competitive level. Rugby code was classified as rugby sevens, rugby league, or rugby union (1). Caffeine dose was classified as low dose (≤3 mg/kg) or moderate dose (>3–6 mg/kg) (9). Forms of supplementation were classified as solid oral administration (capsules/tablets), liquid administration (supplement drinks/energy drinks), or oromucosal administration (caffeine gum/mouth rinse) (18). Competitive level was classified according to the athlete classification framework proposed by McKay et al. Based on this framework, participants in the included studies were mainly categorized as Tier 2: Developmental, Tier 3: National Level, or Tier 4: International Level (45).

Sensitivity analyses were performed using a leave-one-out approach (35). Funnel plots and Egger’s regression tests were used, where appropriate, to explore potential small-study effects or publication bias (46). Given the limited number of independent studies for several outcomes and the presence of multiple dependent effect sizes from the same studies, the results of publication bias assessments were interpreted cautiously and considered exploratory. All statistical tests were two-sided, and *p* < 0.05 was considered statistically significant.

## 3 Results

### 3.1 Literature selection process

**Figure 1:**
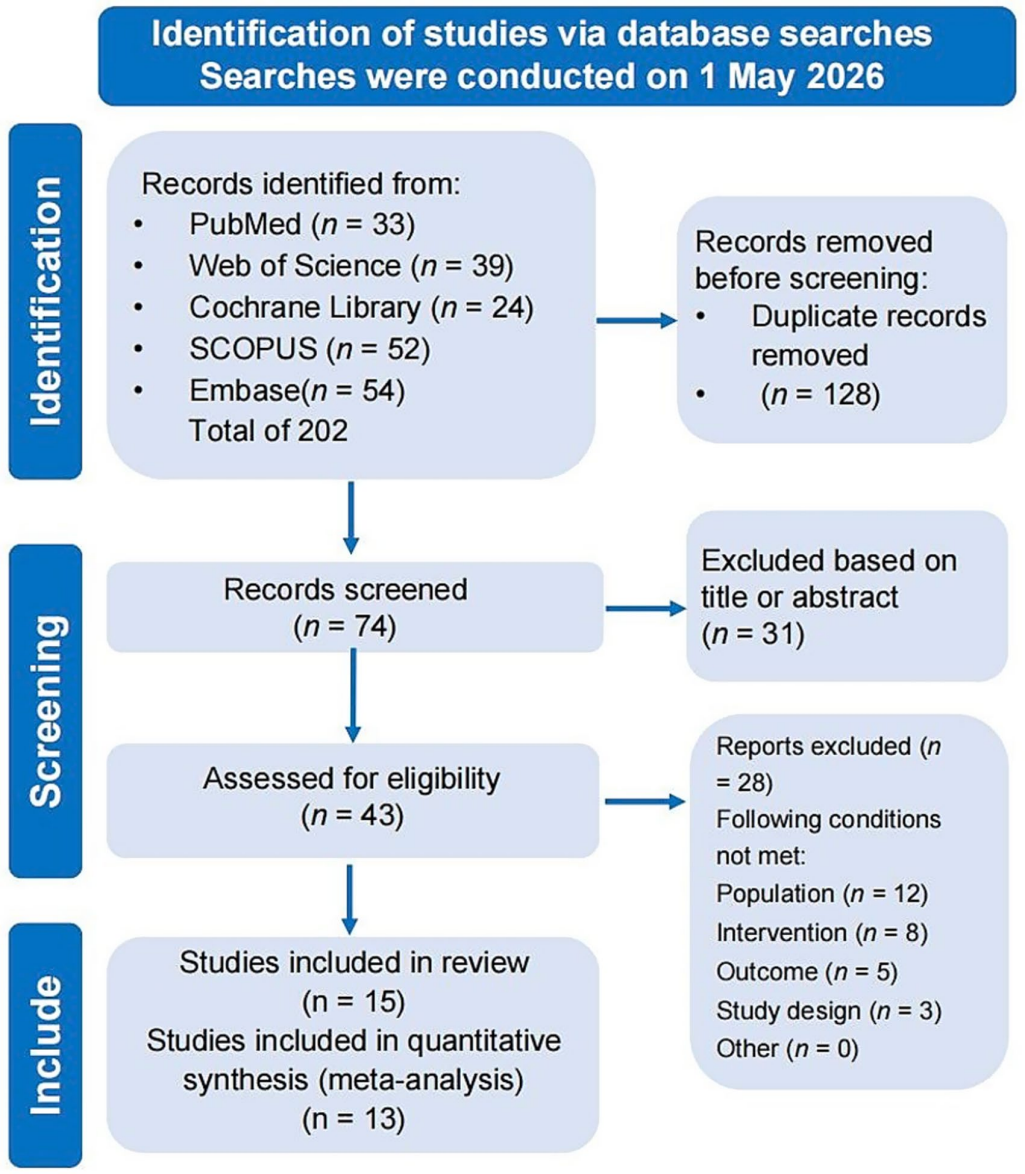
PRISMA flow diagram of the study selection process.

Database searches identified 202 records, including PubMed (*n* = 33), Web of Science (*n* = 39), Cochrane Library (*n* = 24), Scopus (*n* = 52), and Embase (*n* = 54). After removal of 128 duplicates, 74 records underwent title and abstract screening, and 31 records were excluded. Forty-three full-text articles were assessed for eligibility; 28 were excluded because they did not meet the inclusion criteria for population (*n* = 12), intervention (*n* = 8), outcome (*n* = 5), or study design (*n* = 3). Finally, 15 studies were included in this review, including 13 studies in the quantitative synthesis. The study selection process is shown in Figure 1.

### 3.2 Characteristics of included studies

#### 3.2.1 Participant characteristics

A total of 15 studies were included in this review, comprising 13 randomized controlled trials and two observational studies, published between 2005 and 2025 (15–18, 24–30, 47–50). By rugby code, nine studies involved rugby union, three involved rugby league, and three involved rugby sevens. Sample sizes ranged from 6 to 27 participants, with a cumulative study-level sample size of 208 participants. The participants were predominantly male athletes. Only two studies included female athletes, both of which involved Spanish national women’s rugby sevens players. Competitive levels ranged from Tier 2 to Tier 4, including university, semi-professional, professional club, and national team athletes. Overall, the included samples mainly comprised competitive athletes; however, variation was observed in sex, competitive level, rugby code, and sample size.

#### 3.2.2 Caffeine supplementation protocols and dose characteristics

Across the 13 intervention studies, caffeine doses ranged from 1 to 6 mg/kg, with 3 mg/kg being the most used dose. Several studies used absolute doses, including 200 mg, 300 mg, and 400 mg, which were approximately equivalent to 2.3–4.1 mg/kg after conversion. The forms of supplementation were classified into three categories: solid oral administration, liquid beverages, and oromucosal absorption. Solid oral administration mainly included capsules or tablets, liquid forms mainly included beverages or energy drinks, and oromucosal absorption included caffeine gum and mouth rinsing. The two observational studies recorded habitual caffeine intake before or during matches but did not standardize the dose or form of intake. The most common timing of administration was approximately 60 min before competition. Other protocols included supplementation 70–90 min before competition, after warm-up, during a simulated half-time interval, or short-term mouth rinsing before exercise. In terms of study design, most studies compared caffeine alone with placebo, whereas four delivered caffeine in carbohydrate-containing or multi-ingredient beverages with matched caffeine-free vehicles as controls (25, 28, 29, 47). These differences suggest a degree of clinical and methodological heterogeneity across intervention protocols (Table 1).

**Table 1:** Basic characteristics of the included studies.

Study	Rugby code	Study design	Region	Participant characteristics	Washout period	Habitual	Caffeine dose	Mode of administration	Timing of administration (min)	Main outcomes
Stuart et al. (26)	Rugby union	RCT	New Zealand	9 competitive male rugby union players; Developmental; Age, 25 ± 4 yr.; Height, 181 ± 4 cm; Body mass, 98 ± 22 kg	7 days	NR	6 mg/kg	Solid oral (capsules)	70	20-m sprint, 30-m sprint; Passing accuracy; Plasma epinephrine
Roberts et al. (28)	Rugby union	RCT	UK	8 male rugby union forwards; Developmental; Age, 22.0 ± 3.5 yr.; Height, 182 ± 3 cm; Body mass, 92.2 ± 14.0 kg	6–14 days	<100 mg/day	4 mg/kg	Liquid (beverages)	60	15-m sprint; Motor skills test; RPE during rugby-specific intermittent running; Blood glucose; Free fatty acids
Cook et al. (48)	Rugby union	RCT	UK	10 elite rugby players; National Level; Age, 20 ± 0.5 yr.; Height, 181 ± 2 cm; Body mass, 90 ± 4 kg	3 days	NR	1 or 5 mg/kg	Solid oral (capsules)	90	Passing accuracy; Salivary testosterone; Salivary cortisol
Del Coso et al. (25)	Rugby sevens	RCT	Spain	16 Spanish national female rugby sevens players; International Level; Age, 23 ± 2 yr.; Height, 166 ± 7 cm; Body mass, 66 ± 7 kg	3 days	<60 mg/day	3 mg/kg	Liquid (beverages)	60	Maximal running speed during the 6 × 30 m sprint test; Pace at sprint velocity during matches; Jump power; RPE during repeated-sprint and match play
Assi et al. (30)	Rugby union	RCT	UK	9 amateur male rugby union players; Developmental; Age, 22.4 ± 1.8 yr.; Height, 180 ± 1 cm; Body mass, 81.7 ± 9 kg	7 days	<300 mg/day	6 mg/kg	Liquid (beverages)	60	Passing accuracy test total score, Left-hand score, right-hand score; RPE during a 40-min simulated rugby protocol
Wellington et al. (24)	Rugby league	RCT	Australia	11 semi-professional male rugby league players; National Level; Age, 19.0 ± 0.5 yr.; Height, 178.9 ± 2.6 cm; Body mass, 87.4 ± 12.9 kg	7 days	NR	3.43 mg/kg (300 mg)	Solid oral (tablets)	60	Total time to complete 9 sprints; Blood lactate
Portillo et al. (47)	Rugby sevens	RCT	Spain	16 Spanish national female rugby sevens players; International Level; Age, 23 ± 2 yr.; Height, 166 ± 7 cm; Body mass, 66 ± 7 kg	3 days	<60 mg/day	3 mg/kg	Liquid (beverages)	60	Technical action frequency; Technical action quality
Dunican et al. (50)	Rugby union	Observational study	Australia	20 professional male Super Rugby players; International Level; Age, 26 ± 3 yr.; Height, 185 ± 7 cm; Body mass, 102 ± 12 kg	NR	NR	NR	NR	49 ± 61	Sleep-related recovery outcomes; Salivary caffeine concentration
Clarke et al. (29)	Rugby league	RCT	UK	8 elite male rugby league forwards from an English Super League club; International Level; Age, 21.4 ± 2.4 yr.; Height, 189.2 ± 7.2 cm; Body mass, 94.9 ± 11.4 kg	7 days	NR	3 mg/kg	Liquid (beverages)	60	Mean sprint speed bout 2; CMJ height; RPE during simulated rugby league match play
Ranchordas et al. (15)	Rugby union	RCT	UK	17 university-standard male rugby union players; Developmental; Age, 20.4 ± 1.2 yr.; Height, 179.4 ± 6.2 cm; Body mass, 85.6 ± 6.3 kg	7 days	NR	2.1 mg/kg (200 mg)	Oromucosal (gum)	Immediately after the warm-up	Fatigue index of 6 × 30 m repeated sprint; CMJ height; Illinois agility test; Blood lactate
Russell et al. (27)	Rugby union	RCT	UK	14 professional academy male rugby union players; National Level; Age, 18 ± 1 yr.; Height, 183 ± 7 cm; Body mass, 98.6 ± 10.9 kg	6–14 days	191 ± 138 mg/day	4.14 mg/kg (400 mg)	Oromucosal (gum)	At the start of the simulated half-time	6 × 40 m repeated sprint test; Salivary testosterone; Salivary cortisol
Caia et al. (49)	Rugby league	Observational study	Australia	15 professional male rugby league players; International Level; Age, 23.0 ± 3.6 yr.; Height, 183 ± 6 cm; Body mass, 99.6 ± 11.3 kg	NR	NR	NR	NR	60	Sleep-related recovery outcomes; Salivary caffeine concentration
Tamilio et al. (17)	Rugby union	RCT	UK	22 university-standard male rugby union players; Developmental; Age 20 ± 2 yr.; Height 181 ± 7 cm; Body mass 91 ± 23 kg	NR	118 ± 88 mg/day	3 mg/kg	Solid oral (capsules)	45	CMJ height; SJ height; IMTP Peak Force; Squat repetitions until failure; RPE after resistance exercise to failure
Tallis et al. (18)	Rugby union	RCT	UK	27 male university rugby union players; Developmental; Age 20 ± 2 yr.; Height 182.0 ± 8.2 cm; Body mass 96.6 ± 18.2 kg	3–5 days	188 ± 88 mg/day	3 mg/kg	Solid oral (capsules)/ Oromucosal (gum/mouth rinse)	Cap: 45; gum: 10; mouth rinse: 1	CMJ height; DJ height; IMTP Peak Force; Squat repetitions until failure; RPE after resistance exercise to failure
Hsueh et al. (16)	Rugby sevens	RCT	Taiwan	6 Division I male collegiate rugby sevens players; National Level; Age 21.5 ± 0.8 yr.; Height 178 ± 9 cm; Body mass 81.3 ± 9.2 kg	13 days	NR	3 mg/kg	Solid oral (capsules)	Before each match	Distance at >20 km/h; Reactive agility

RCT, randomized controlled trial; RPE, rating of perceived exertion; FFA, free fatty acids; PAT, passing accuracy test; RHIE, repeated high-intensity effort; CMJ, countermovement jump; SJ, squat jump; DJ, drop jump; IMTP, isometric mid-thigh pull; RSSA, repeated sprint ability test; IR2, intermittent recovery test level 2; NR: not reported; yr: years.

### 3.3 Quality assessment of included studies

The methodological quality of the 13 randomized placebo-controlled trials was assessed using the PEDro scale, and the results are presented in Supplementary Table 3. PEDro scores ranged from 7 to 10, with a mean score of 8.0, indicating generally high methodological quality. Most studies were rated as good to excellent. The main sources of score deduction were allocation concealment, assessor blinding, complete follow-up, and intention-to-treat analysis.

**Figure 2:**
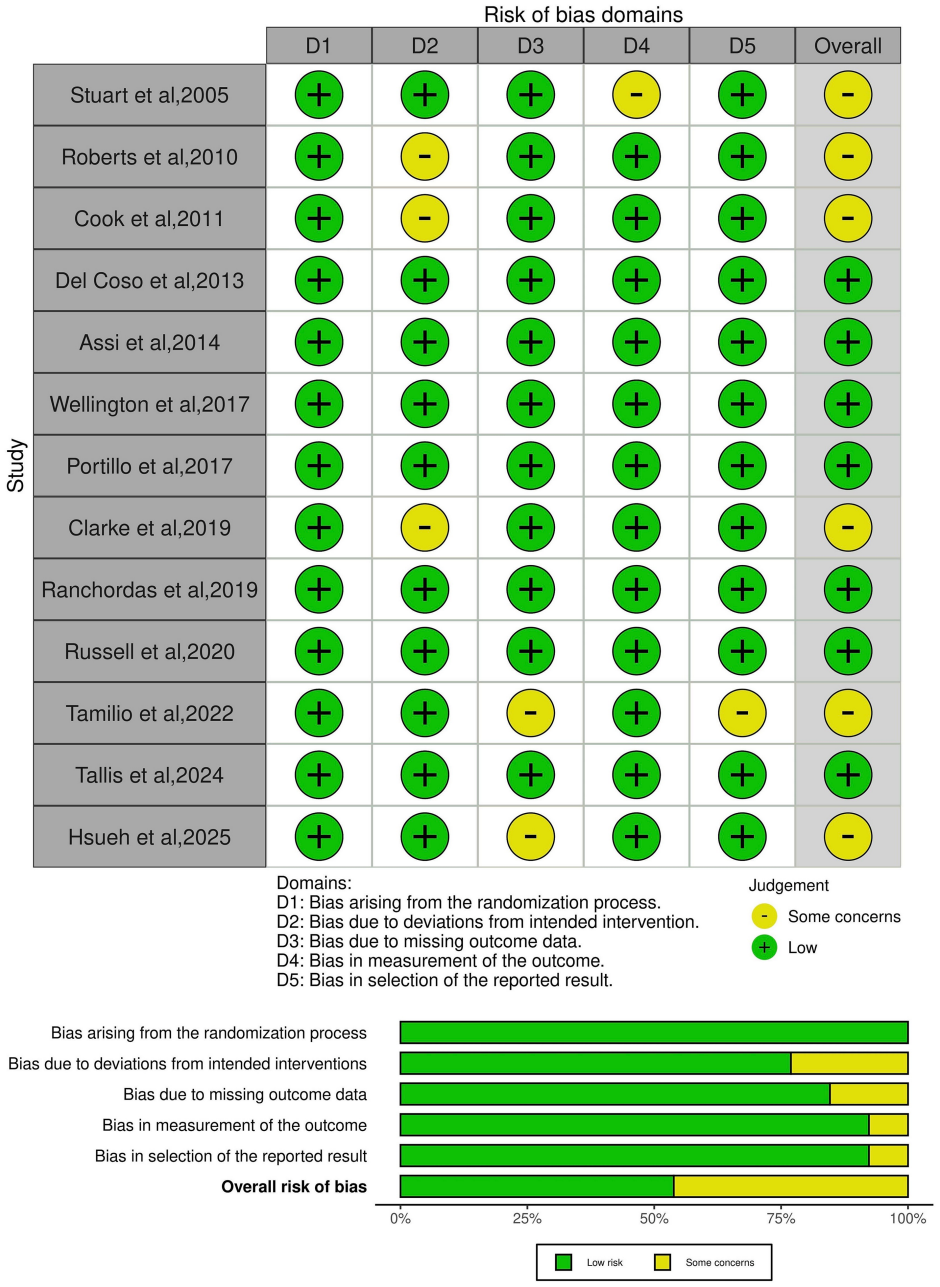
Risk of bias results.

The RoB 2 assessment showed that the overall risk of bias in the included intervention studies was low, with no study judged as having a high risk of bias (Figure 2). Seven studies were rated as having an overall low risk of bias, whereas six studies raised some concerns. The domains with some concerns mainly involved deviations from intended interventions, outcome measurement, missing outcome data, and selection of the reported result.

The two sleep-related observational studies were assessed using the JBI Critical Appraisal Checklist for Analytical Cross-Sectional Studies, and both scored 7 out of 8, as shown in Supplementary Table 4. Both studies assessed sleep using wrist-worn activity monitors and measured caffeine concentrations using saliva samples. The main limitation was insufficient control for potential confounding factors. Therefore, these observational studies were used only for supplementary narrative synthesis of sleep- and recovery-related outcomes and were not included in the main effect-size pooling or the GRADE certainty assessment.

### 3.4 Meta-analysis results

#### 3.4.1 Physical performance

**Figure 3:**
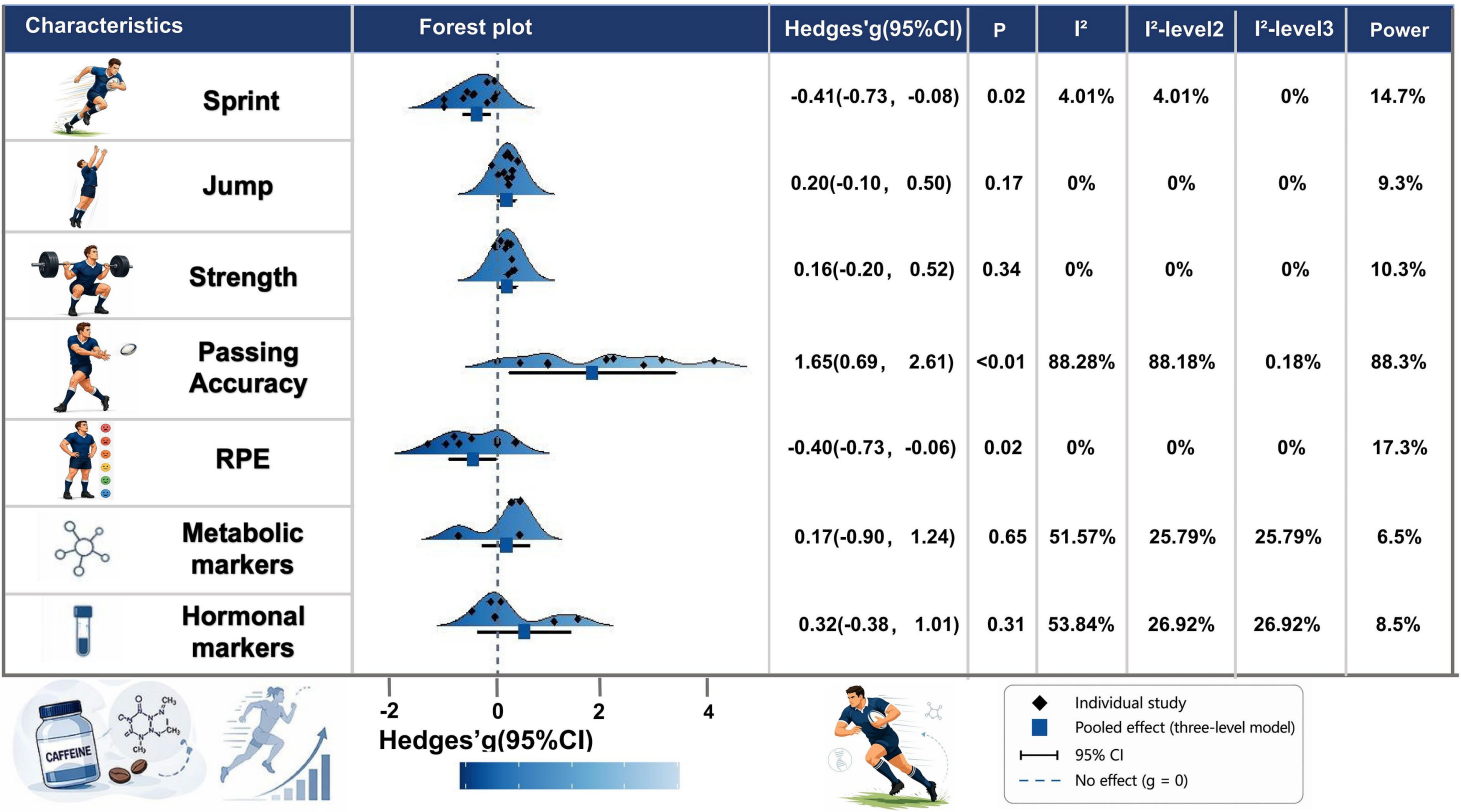
Primary pooled effect sizes for the main outcomes. 95% CI, 95% confidence interval; Hedges’ *g*, effect size used in the pooled analysis; I^2^, indicator of heterogeneity; P, *p*-value for the pooled effect; Power, statistical power for the pooled effect size.

Because sprint-related outcomes were mostly time- or time-related measures, negative effect sizes indicated improved performance. The primary analysis showed that, compared with placebo, acute caffeine supplementation was associated with improved sprint performance, with a small effect size (*k* = 11, Hedges’ *g* = −0.41, 95% CI: −0.73 to −0.08, *I*^2^ = 4.01%, *I*^2^-level 2 = 4.01%, *I*^2^-level 3 = 0%, *p* = 0.02; Figure 3). In contrast, acute caffeine supplementation showed no statistically significant effects on jumping performance (*k* = 11, Hedges’ *g* = 0.20, 95% CI: −0.10 to 0.50, *I*^2^ = 0%, *I*^2^-level 2 = 0%, *I*^2^-level 3 = 0%, *p* = 0.17) or strength-related performance (*k* = 10, Hedges’ *g* = 0.16, 95% CI: −0.20 to 0.52, *I*^2^ = 0%, *I*^2^-level 2 = 0%, *I*^2^-level 3 = 0%, *p* = 0.34).

#### 3.4.2 Sport-specific technical performance

The primary analysis showed that acute caffeine supplementation was associated with improved passing accuracy (*k* = 9, Hedges’ *g* = 1.65, 95% CI: 0.69 to 2.61, *I*^2^ = 88.28%, *I*^2^-level 2 = 88.1%, *I*^2^-level 3 = 0.18%, *p* = 0.004; Figure 3). However, this outcome showed high heterogeneity, and publication bias assessment suggested a possible small-study effect. Therefore, this finding should be interpreted with caution.

In addition to passing accuracy, Portillo et al. (47). assessed the frequency and quality of technical actions in real international women’s rugby sevens match settings. This study found that pre-match ingestion of a 3 mg/kg caffeinated energy drink had no significant effect on the frequency or quality ratings of technical actions, including tackles, rucks, passes, pass receptions, and ball carries.

#### 3.4.3 Perceptual responses

The pooled analysis of RPE measured during or immediately after rugby-specific intermittent or match-play protocols and resistance-exercise repetitions-to-failure tests showed that acute caffeine supplementation was associated with lower RPE than the control condition (*k* = 9, Hedges’ *g* = −0.40, 95% CI: −0.73 to −0.06, *I*^2^ = 0%, *I*^2^-level 2 = 0%, *I*^2^-level 3 = 0%, *p* = 0.02; Figure 3).

#### 3.4.4 Physiological markers

##### 3.4.4.1 Metabolic and hormonal markers

For metabolic and hormonal markers (Figure 3), acute caffeine supplementation did not significantly alter metabolic outcomes (*k* = 4, Hedges’ *g* = 0.17, 95% CI: −0.90 to 1.24, *I*^2^ = 51.57%, *I*^2^-level 2 = 25.79%, *I*^2^-level 3 = 25.79%, *p* = 0.65) or hormonal outcomes (*k* = 7, Hedges’ *g* = 0.32, 95% CI: −0.38 to 1.01, *I*^2^ = 53.84%, *I*^2^-level 2 = 26.92%, *I*^2^-level 3 = 26.92%, *p* = 0.31).

Detailed pooled forest plots for each outcome are provided in Supplementary Figures 1–7.

Additionally, a visual plot of statistical power for the pooled results of all outcomes can be found in Supplementary Figures 8–14.

##### 3.4.4.2 Sleep- and recovery-related outcomes from observational evidence

Observational studies suggested that caffeine exposure on match day or after competition may be associated with impaired sleep recovery. Dunican et al. (50) and Caia et al. (49) both observed delayed bedtime, reduced sleep duration, and increased sleep latency on the night after competition, which were associated with elevated post-match salivary caffeine concentrations. In addition, Del Coso et al. (25) reported a higher incidence of insomnia and gastrointestinal discomfort after caffeine ingestion, whereas headache and anxiety were not clearly increased. Under sleep-deprivation conditions, Cook et al. (48) observed increased salivary cortisol after caffeine intake, but no significant change in testosterone (Table 2).

**Table 2:** Summary of sleep, recovery, and adverse-effect outcomes associated with caffeine exposure in rugby players.

**Study**	**Participants/context**	**Adverse or recovery-related outcomes**	**Main Findings**	**Overall interpretation**
Cook et al. (48)	10 elite rugby players; sleep-deprivation condition	Salivary cortisol, salivary testosterone	A 5 mg/kg caffeine dose increased salivary cortisol compared with placebo, whereas salivary testosterone showed no significant change.	Suggests a possible increase in stress response at higher caffeine doses
Del Coso et al. (25)	16 female rugby sevens players; side-effect survey conducted hours after competition	Headache, abdominal/gut discomfort, muscle soreness, insomnia, and related symptoms	Insomnia was more frequent after the caffeinated energy drink than after placebo (63% vs. 19%). Abdominal/gut discomfort was also more common (25% vs. 6%), whereas headache and muscle soreness were not increased.	Adverse effects were mainly related to insomnia and gastrointestinal discomfort
Dunican et al. (50)	20 elite rugby union players; evening Super Rugby match	Bedtime, sleep duration, sleep latency, sleep efficiency, salivary caffeine concentration	Bedtime was delayed by ~3 h and sleep duration was reduced by ~1.5 h on the night of competition. Post-match salivary caffeine concentration was higher than pre-match in most players and was associated with longer sleep latency and lower sleep efficiency.	Suggests impaired post-match sleep and recovery
Caia et al. (49)	15 professional rugby league players; sleep monitored before, on, and after competition night	Bedtime, sleep duration, salivary caffeine concentration	On the night of competition, players went to bed later and had shorter sleep duration, while post-match salivary caffeine concentration was markedly elevated. Increases in caffeine concentration were directionally associated with longer sleep latency and lower sleep efficiency.	Suggests post-competition sleep disturbance

### 3.5 Moderator analysis

We further examined the potential moderating effects of rugby code, caffeine dose, form of supplementation, and competitive level on the main outcomes. Overall, most between-subgroup differences did not reach statistical significance, suggesting that current evidence is insufficient to confirm a clear moderating effect of these factors on the ergogenic effects of caffeine. Because some subgroups included only one or two effect sizes, the following findings should be considered exploratory.

For sprint performance, subgroup analysis showed an improvement under the low-dose caffeine condition (Hedges’ *g* = −0.50, 95% CI: −0.92 to −0.17, *p* = 0.01; Table 3). However, the between-dose subgroup difference was not statistically significant (*p*_between = 0.19).

**Table 3:** Subgroup analyses for sprint performance.

**Subgroup**	***K***	***N***	**Hedges’ *g***	**95% CI**	***P***_**d**_	**Power**	***P***_**b**_
Rugby code							0.73
Rugby sevens	3	76	−0.25	[−0.81, 0.28]	0.31	19.3%	
Rugby league	3	54	−0.55	[−1.21, 0.10]	0.08	49.8%	
Rugby union	5	118	−0.40	[−0.84, 0.04]	0.06	55.7%	
Dose							0.19
Low	6	142	−0.55	[−0.94, −0.16]	0.01	89%	
Moderate	5	106	−0.18	[−0.62, 0.25]	0.36	15.9%	
Supplement form							0.61
Solid oral	4	74	−0.20	[−0.74, 0.33]	0.41	14%	
Liquid	5	112	−0.43	[−0.88, 0.01]	0.05	60.7%	
Oromucosal	2	62	−0.54	[−1.16, 0.06]	0.07	54.5%	
Competitive level							0.61
T2	4	90	−0.52	[−1.02, −0.02]	0.04	67.3%	
T3	3	62	−0.18	[−0.77, 0.40]	0.49	11%	
T4	4	96	−0.40	[−0.88, 0.07]	0.08	50.1%	

K, number of effect sizes included in the subgroup analysis; N, sum of condition-level sample sizes included in the model rather than the number of independent participants; Hedges’ g, effect size used in the pooled analysis; 95% CI, 95% confidence interval; Pd, *p*-value for the pooled effect within each subgroup; Power, statistical power for the pooled effect size; Pb, *p*-value for between-subgroup differences.

For the pooled exercise-RPE outcome, reductions were observed in the solid oral administration subgroup and among Tier 4 athletes, but between-subgroup differences were not statistically significant (Table 4).

**Table 4:** Subgroup analyses for RPE.

**Subgroup**	***K***	***N***	**Hedges’ *g***	**95% CI**	***P***_**d**_	**Power**	***P***_**b**_
Rugby code							0.26
Rugby league	2	32	−1.06	[−2.12, 0.01]	0.05	68.2%	
Rugby sevens	1	32	−0.48	[−1.63, 0.66]	0.33	18.0%	
Rugby union	6	240	−0.21	[−0.66, 0.24]	0.31	20.1%	
Dose							0.63
Low	7	270	−0.41	[−0.87, 0.05]	0.07	55.1%	
Moderate	2	34	−0.16	[−1.21, 0.87]	0.71	6.7%	
Supplement form							0.05
Liquid	5	210	−0.12	[−0.45, 0.20]	0.41	14.8%	
Solid oral	4	94	−0.73	[−1.24, −0.21]	0.01	92.1%	
Competitive level							0.15
T2	6	240	−0.21	[−0.63, 0.22]	0.29	20.5%	
T4	3	64	−0.79	[−1.54, −0.05]	0.04	70.9%	

K, number of effect sizes included in the subgroup analysis; N, sum of condition-level sample sizes included in the model rather than the number of independent participants; Hedges’ *g*, effect size used in the pooled analysis; 95% CI, 95% confidence interval; Pd, *p*-value for the pooled effect within each subgroup; Power, statistical power for the pooled effect size; Pb, *p*-value for between-subgroup differences.

For passing accuracy, improvements were observed under both low- and moderate-dose caffeine conditions, and liquid supplementation also showed a positive effect (Table 5). However, given the high heterogeneity and potential small-study effect for this outcome, these subgroup findings should be interpreted with caution. Regarding competitive level, passing accuracy improved in both Tier 2 and Tier 3 athletes, with a larger effect size observed in Tier 3 athletes; however, the between-group difference did not reach conventional statistical significance (*p*_between = 0.07).

**Table 5:** Subgroup analyses for passing accuracy.

**Subgroup**	***K***	***N***	**Hedges’ *g***	**95% CI**	***P***_**d**_	**Power**	***P***_**b**_
Dose							0.54
Low	2	40	2.41	[0.19, 4.61]	0.03	73.5%	
Moderate	7	128	1.55	[0.38, 2.75]	0.01	88.2%	
Supplement form							0.22
Solid oral	4	70	1.32	[−0.21, 2.85]	0.08	53.1%	
Liquid	5	98	2.07	[0.7, 3.44]	<0.01	94.8%	
Competitive level							0.07
T2	5	88	1.05	[0.00, 2.12]	0.04	66%	
T3	4	80	2.51	[1.42, 3.58]	<0.01	99.8%	

K, number of effect sizes included in the subgroup analysis; N, sum of condition-level sample sizes included in the model rather than the number of independent participants; Hedges’ *g*, effect size used in the pooled analysis; 95% CI, 95% confidence interval; Pd, *p*-value for the pooled effect within each subgroup; Power, statistical power for the pooled effect size; Pb, *p*-value for between-subgroup differences.

### 3.6 Publication bias assessment

**Figure 4:**
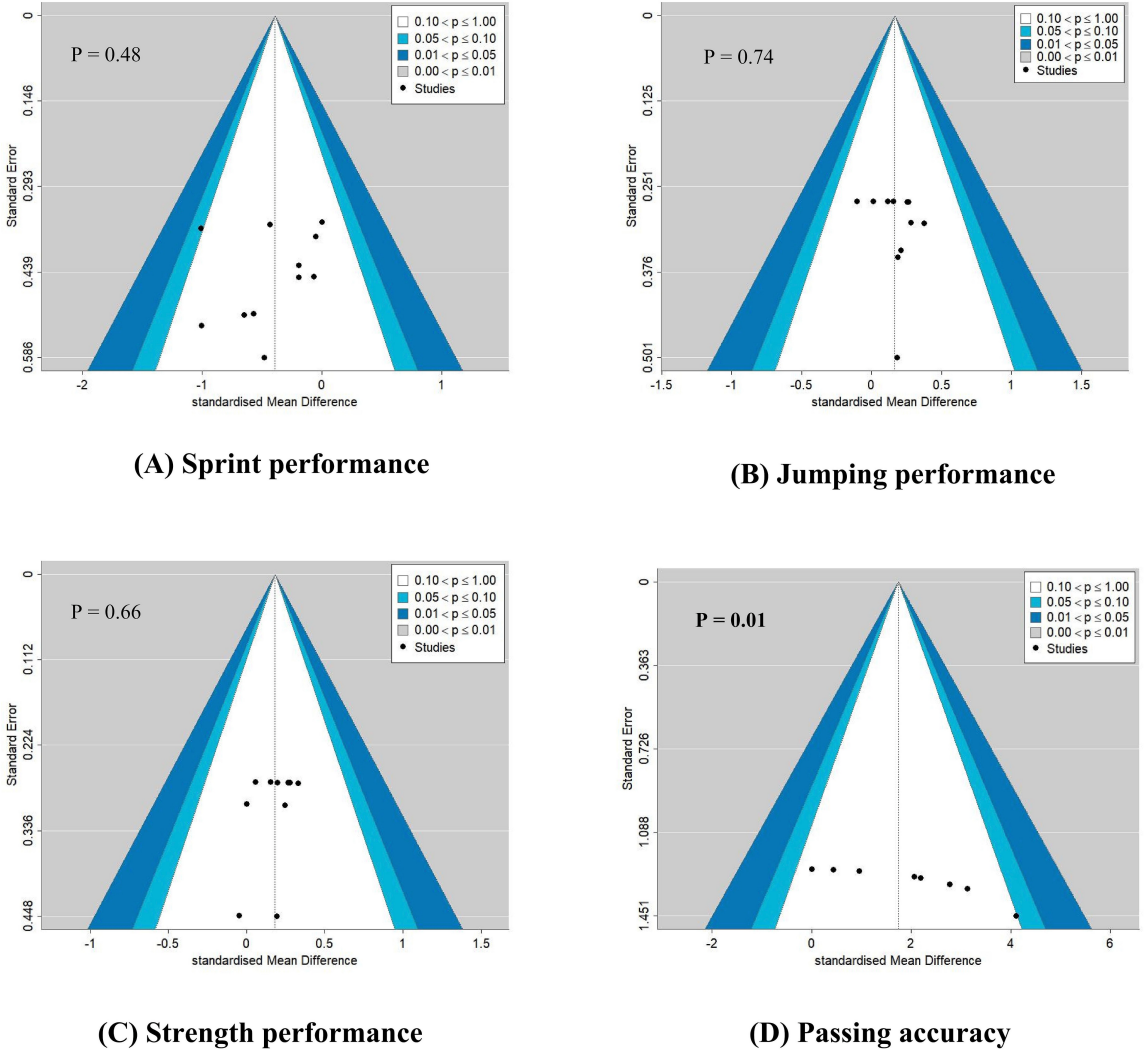
Contour-enhanced funnel plots for small-study effects in **(A)** sprint performance, **(B)** jumping performance, **(C)** strength performance, and **(D)** passing accuracy. Egger’s test was significant only for passing accuracy (*p* = 0.01), suggesting potential funnel plot asymmetry.

Funnel plots and Egger’s regression tests were used to conduct exploratory assessments of potential small-study effects for sprint performance, jumping performance, strength performance, and passing accuracy (Figure 4). Egger’s tests showed no statistical evidence of obvious small-study effects for sprint performance (*p* = 0.48), jumping performance (*p* = 0.74), or strength performance (*p* = 0.66). For passing accuracy, the exploratory Egger’s test was statistically significant (*p* = 0.01), and the funnel plot displayed visual asymmetry. This asymmetry may reflect several factors, including but not limited to small-study effects, genuine heterogeneity in effects, or the methodological artifact of including multiple dependent effect sizes from the same studies in the funnel plot. Given the severely limited number of studies, these findings cannot distinguish between these possibilities and do not provide credible evidence of publication bias. Nevertheless, this observation aligns with the previously noted high heterogeneity and reinforces the need for cautious interpretation of the pooled effect for passing accuracy.

### 3.7 Sensitivity analysis and influence diagnostics

#### 3.7.1 Sensitivity analysis

The conventional leave-one-out analysis showed that the direction of the pooled effect for sprint performance remained generally consistent (Supplementary Figures 15–21). After removing any single study or effect size, the effect remained negative, indicating improved sprint performance (Hedges’ *g* = −0.301 to −0.450, *p* ≤ 0.03). Passing accuracy also maintained a large positive effect in the leave-one-out analysis (Hedges’ *g* = 1.51 to 1.98, *p* < 0.01). In contrast, the direction of effects for strength performance and jumping performance was generally consistent, but statistical significance was unstable. The direction of the pooled exercise-RPE effect remained negative in the leave-one-out analysis, although statistical significance was lost in some removal scenarios. Metabolic and hormonal markers remained non-significant after the removal of individual studies or effect sizes.

#### 3.7.2 Influence diagnostics

Influence diagnostics identified no clear outlying effect sizes for jumping performance, strength performance, or metabolic markers. Potential influential effect sizes were detected for sprint performance, passing accuracy, RPE, and hormonal markers. Sprint performance was relatively sensitive to the effect size from Ranchordas et al. (15). After removing this effect size or study, the direction of effect still supported a beneficial effect of caffeine on sprint performance, but the statistical significance was attenuated (Hedges’ *g* = −0.29, *p* = 0.099). For passing accuracy, two potentially influential effect sizes were identified at the effect-size level, from Stuart et al. and Roberts et al.; however, no clear outlying study was detected at the study level. After excluding these potentially influential effect sizes, the pooled effect remained positive but was no longer statistically significant (Hedges’ *g* = 1.49, 95% CI: −0.71 to 3.69, *p* = 0.149), suggesting that the evidence for passing accuracy was sensitive to individual effect sizes and should be interpreted cautiously. Influence diagnostics also suggested limited robustness for the pooled exercise-RPE outcome and hormonal markers.

### 3.8 Certainty of evidence

**Figure 5:**
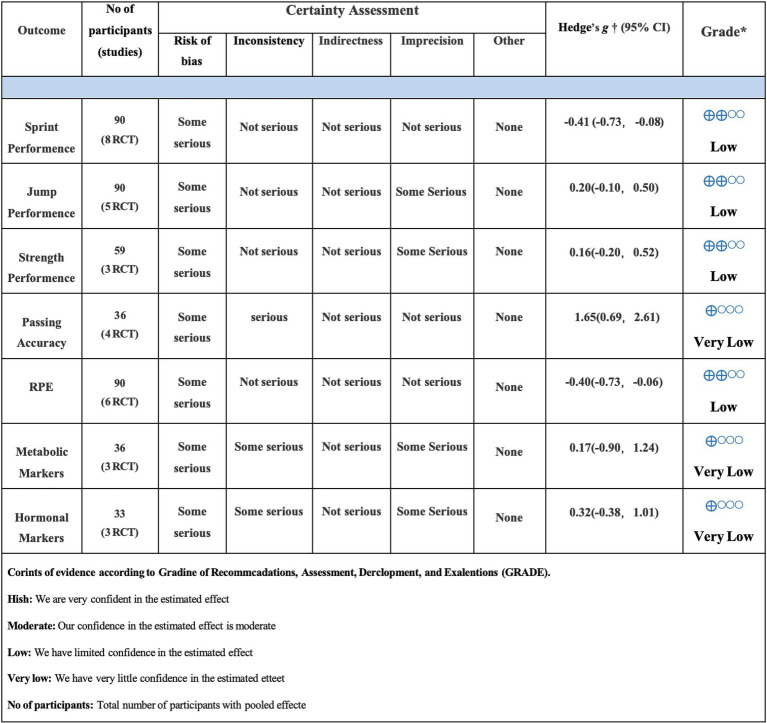
GRADE assessment of the certainty of evidence for the main outcomes.

The certainty of evidence for the main outcomes was assessed using the GRADE framework (Figure 5). Overall, the certainty of evidence for the included outcomes ranged from low to very low. The certainty of evidence was rated as low for sprint performance, jumping performance, strength performance, and the pooled exercise-RPE outcome, and very low for passing accuracy, metabolic markers, and hormonal markers. The main reasons for downgrading were risk of bias, inconsistency, and imprecision. For passing accuracy, the certainty of evidence was additionally affected by heterogeneity and potential small-study effects or publication bias.

## 4 Discussion

This meta-analysis indicated that acute caffeine supplementation had limited beneficial effects on selected rugby-related outcomes. Small improvements were observed for sprint performance, together with lower RPE measured during or immediately after rugby-specific or resistance-exercise protocols, whereas no consistent effects were found for jumping performance, strength performance, metabolic markers, or hormonal markers. Although a large positive pooled effect was observed for passing accuracy in standardized tests, this outcome showed high heterogeneity, potential small-study effects or publication bias, and very low certainty of evidence according to GRADE. Therefore, this finding should not be interpreted as evidence that caffeine consistently improves sport-specific technical performance in real match settings.

Taken together, the present findings are better interpreted as suggesting that acute caffeine supplementation may provide limited benefits in certain rugby-related tasks, particularly short-duration high-intensity running, standardized passing tests, and RPE. However, current evidence is insufficient to support broad beneficial effects of caffeine on physical performance, sport-specific technical performance, perceptual responses, or physiological markers in rugby players. These findings should be discussed in light of the small sample sizes of the included studies, dependence among effect sizes, differences in testing contexts, sensitivity analysis results, and the certainty of evidence.

### 4.1 Effects of acute caffeine supplementation on physical performance

The present study showed that acute caffeine supplementation was associated with a small improvement in sprint performance in rugby players. This finding is generally consistent with previous systematic reviews on repeated sprint ability, suggesting that caffeine may be beneficial for short-duration, high-intensity, and repeated running tasks (10). In rugby, sprinting usually occurs in contexts involving repeated high-intensity intermittent running, physical contact, and brief recovery periods (51). These demands are likely to be influenced by central nervous system excitability, perceived fatigue, and neuromuscular output. Therefore, caffeine may help players maintain sprint output under accumulated fatigue by antagonizing adenosine receptors, increasing alertness, and reducing perceived exertion (52). Sprint-related measures may be particularly sensitive to these central and perceptual effects because they often require athletes to maintain output across repeated high-intensity efforts under accumulated fatigue, whereas isolated jumping and strength tests may be more strongly constrained by baseline neuromuscular capacity, technical execution, and test-specific variability (52). However, the sprint finding should be interpreted with caution. First, the pooled effect size for sprint performance was small, indicating that the practical benefit may be limited. Second, sensitivity analysis showed that the sprint result was relatively sensitive to the effect size from Ranchordas et al. (15). After removing this effect size or study, the direction of the effect still supported a beneficial effect of caffeine on sprint performance, but the statistical significance was attenuated. Therefore, the current findings are more appropriately interpreted as suggesting that acute caffeine supplementation may provide a small benefit for sprint performance.

Compared with sprint performance, the present study did not observe consistent significant effects of acute caffeine supplementation on jumping or strength performance. This finding is not entirely consistent with the systematic review and meta-analysis by Chen et al. on volleyball players (11). Chen et al. reported that caffeine improved general physical performance measures such as vertical jump and handgrip strength, but did not significantly affect volleyball-specific jumping tasks such as attack jump and block jump. Several explanations may account for this discrepancy. The included primary studies used heterogeneous assessments, including the countermovement jump, squat jump, drop jump, isometric mid-thigh pull, and repetitions to failure in the squat or bench press (17, 18). These tests represent different neuromuscular qualities and may vary in their sensitivity to acute caffeine supplementation. In addition, strength-, speed-, and power-related performance can vary between and within elite rugby players across a competitive season (53). Rugby players generally have relatively high baseline strength and explosive power, which may limit the additional improvement achievable through an acute nutritional intervention. Based on the current findings, caffeine cannot yet be considered to consistently improve jumping or strength performance in rugby players.

### 4.2 Effects of acute caffeine supplementation on sport-specific technical performance

The present study observed a large positive pooled effect for passing accuracy in standardized passing tests. This finding is consistent with earlier rugby simulation research, in which Stuart et al. reported that 6 mg/kg caffeine improved passing accuracy after high-intensity simulated exercise (26). Passing performance depends not only on technical execution but also on visual search, attentional allocation, reaction speed, decision-making, and motor control (54). Therefore, the effects of caffeine on increasing alertness, reducing perceived fatigue, and maintaining neuromuscular output may help players preserve passing quality after high-intensity exercise. However, although the effect size for passing accuracy was large, this outcome showed high heterogeneity, and Egger’s test suggested possible small-study effects or publication bias. In addition, sensitivity analysis showed that after removing the potentially influential effect size from Stuart et al., the positive effect remained, but its statistical significance decreased to a borderline level.

Moreover, findings from standardized passing tests may not be fully generalizable to real match play. Technical performance in actual competition is jointly influenced by defensive pressure, tactical role, teammate coordination, ball possession opportunities, match tempo, and decision-making complexity (47, 54). As shown in the results, Portillo et al. (47) found no significant effect of caffeine on the frequency or quality ratings of technical actions, including tackles, rucks, passes, pass receptions, and ball carries, in real international women’s rugby sevens matches. Therefore, the current evidence is more appropriately interpreted as suggesting that caffeine may improve performance in some standardized or semi-standardized passing tests, rather than demonstrating a consistent enhancement of overall sport-specific technical quality in real match settings.

### 4.3 Effects of acute caffeine supplementation on perceptual responses and physiological markers

The present study showed that acute caffeine supplementation was associated with reduced RPE, which is not entirely consistent with the meta-analysis by Zhang et al. on female basketball players (55). That study reported no significant effect of 2.1–9 mg/kg caffeine on RPE. This discrepancy may be related to differences in sport-specific demands. Rugby involves more frequent physical contact and collision loads, and RPE may therefore be more sensitive to changes in pain perception and central fatigue. However, this finding should still be interpreted with caution. Although RPE was significantly reduced in the primary analysis, sensitivity analysis suggested limited robustness, as statistical significance was lost in some removal scenarios. Therefore, lower RPE may represent one possible pathway underlying the potential performance benefits of caffeine, although current evidence is insufficient to confirm a stable fatigue-perception-modulating effect in rugby players.

For physiological markers, the present study found no consistent significant effects of acute caffeine supplementation on blood lactate, blood glucose, free fatty acids, plasma epinephrine, salivary testosterone, or salivary cortisol. These markers are easily influenced by exercise load, sampling time, dietary control, sleep status, and psychological stress (56). In addition, metabolic and hormonal markers do not have a uniform direction of “improvement.” For example, increased blood lactate may reflect greater glycolytic contribution or exercise output, whereas increased cortisol may indicate an enhanced stress response (57, 58). Therefore, the current findings are more appropriately interpreted as showing that acute caffeine supplementation has not demonstrated consistent effects on metabolic or endocrine responses in rugby players.

Beyond performance outcomes, caffeine-related adverse effects and recovery risks should also be considered. Existing observational evidence suggests that caffeine exposure on match day or after competition may be associated with impaired sleep recovery, mainly reflected by delayed bedtime, reduced sleep duration, and increased sleep latency. Some intervention studies have also reported that caffeinated energy drinks may increase the incidence of insomnia and gastrointestinal discomfort. Therefore, the application of caffeine should not focus only on its potential ergogenic effects during competition or testing, but should also consider post-match recovery, sleep quality, and individual tolerance.

### 4.4 Subgroup analysis

The present study further conducted subgroup analyses based on rugby code, caffeine dose, form of supplementation, and competitive level. However, most between-subgroup differences did not reach statistical significance. Therefore, the current findings do not provide evidence that rugby code, dose, supplementation form, or competitive level clearly moderates the effects of caffeine.

Regarding rugby code, rugby union, rugby league, and rugby sevens differ in match duration, running patterns, frequency of physical contact, and tactical demands, all of which may theoretically influence the effects of caffeine. However, the outcome measures used across different rugby codes were not consistent, and the number of available studies was limited. Therefore, it remains unclear whether any specific rugby code is more responsive to caffeine supplementation.

Regarding dose and form of supplementation, both low- and moderate-dose caffeine showed positive effects for some outcomes, but the between-group differences were not statistically significant. Thus, these findings do not support a clear dose–response relationship, nor do they indicate that one form of supplementation is superior to another. Recent primary evidence comparing encapsulated caffeine with caffeine gum also showed substantial interindividual variability in strength and power responses, supporting an individualized rather than form-based approach to supplementation (59). Given that higher caffeine doses may increase the risk of insomnia, gastrointestinal discomfort, palpitations, or anxiety, practical application should place greater emphasis on individual tolerance rather than simply pursuing higher doses.

Regarding competitive level, no clear differences were observed across athlete tiers. Primary studies indicate that baseline physical characteristics and strength–speed–power profiles may differ across competition levels and may also vary within elite rugby players over time (51, 53). However, it remains unclear whether these differences meaningfully influence the acute ergogenic response to caffeine. Because competitive level is often intertwined with rugby code, sex, testing task, and supplementation protocol, its independent moderating effect is difficult to determine at this stage. Overall, the subgroup findings do not support the development of uniform recommendations regarding dose, supplementation form, or target population.

### 4.5 Practical applications

Based on the present findings, acute caffeine supplementation may be considered a potential nutritional strategy for rugby players in specific training or competition contexts, particularly when sprinting, repeated high-intensity running, or maintenance of technical actions under fatigue is required. However, caffeine did not consistently improve jumping performance, strength, metabolic markers, or hormonal markers; therefore, it should not be regarded as a general strategy for enhancing overall rugby performance.

In practice, although the present meta-analysis did not identify a clear dose–response relationship or superiority of any specific supplementation form, approximately 3 mg/kg may be considered a conservative starting dose and adjusted according to individual responses (9, 17). Capsules or beverages are generally suitable for ingestion approximately 45–60 min before competition (17, 25), whereas caffeine gum may be more appropriate before warm-up or when preparation time is limited (15). For athletes competing in evening matches, facing congested schedules, having poor sleep quality, experiencing gastrointestinal sensitivity, or showing strong responses to caffeine, caffeine should be used cautiously or at a reduced dose. Athletes should not try caffeine supplementation for the first time on the day of an official match. Instead, its effects on performance, perceived exertion, sleep quality, and adverse responses should be evaluated in advance during training or simulated competition.

### 4.6 Strengths and limitations

This study has several strengths. First, it focused specifically on rugby rather than pooling rugby with other team sports, which allowed a more targeted evaluation of the effects of acute caffeine supplementation in rugby-specific contexts. Second, this review included physical performance, sport-specific technical performance, perceptual responses, physiological markers, and recovery-related outcomes, providing a relatively comprehensive overview of the effects of caffeine on both external performance and internal responses in rugby players. Third, a three-level random-effects model was used to account for dependence among multiple effect sizes within the same study. In addition, sensitivity analyses, publication bias assessment, and the GRADE approach were used to evaluate the robustness of the findings and the certainty of evidence.

This study also has several limitations. First, the number of included studies was limited, and most had small sample sizes, resulting in insufficient statistical power for some outcomes and subgroup analyses. Second, differences across studies in rugby code, sex, competitive level, caffeine dose, form of supplementation, timing of administration, and testing protocols may have affected the stability of the pooled effects. In studies using carbohydrate-containing or multi-ingredient vehicles, the non-caffeine ingredients were matched between conditions; however, interactions between caffeine and the delivery vehicle cannot be excluded. Third, some outcomes were informed by only a small number of studies, particularly passing accuracy, metabolic markers, hormonal markers, and several subgroup findings; therefore, these conclusions should be interpreted with caution. Fourth, evidence in female athletes remains clearly insufficient, and the existing female samples were mainly drawn from Spanish national women’s rugby sevens players, limiting their representativeness. Fifth, some studies may have involved the same or highly similar athlete cohorts; therefore, the study-level cumulative sample size should not be interpreted simply as the number of fully independent participants. Sixth, sleep-related evidence was mainly derived from observational studies, which precludes causal inference regarding the relationship between caffeine exposure and sleep changes.

Future studies should use larger samples and preregistered, double-blind, randomized, placebo-controlled designs. They should also evaluate the effects of caffeine on running load, physical contact, technical execution, decision-making performance, and post-match recovery in real match settings or highly simulated rugby contexts. In addition, future research should improve the control and reporting of female athlete participation, competitive level, habitual caffeine intake, timing of supplementation, sleep recovery, and individual adverse responses.

## 5 Conclusion

This systematic review and meta-analysis indicated that acute caffeine supplementation may have limited beneficial effects on selected outcomes in rugby players, including a small improvement in sprint performance, a positive effect on standardized passing accuracy, and a possible reduction in RPE measured during or immediately after rugby-specific or resistance-exercise protocols. In contrast, caffeine showed no consistent significant effects on jumping performance, strength performance, metabolic markers, or hormonal markers. It should be noted that the certainty of evidence for sprint performance and RPE was low. Although passing accuracy showed a large effect, this outcome was affected by heterogeneity, potential publication bias, and very low certainty of evidence. Subgroup analyses did not identify clear moderating effects of rugby code, caffeine dose, form of supplementation, or competitive level. In practice, caffeine supplementation should be individualized according to athletes’ responses, match demands, timing of administration, sleep requirements, and risk of adverse effects. Current evidence remains limited, and further high-quality studies are needed to confirm its effects, safety, and recovery-related implications in real rugby match settings.

## Data Availability

The original contributions presented in the study are included in the article/Supplementary material, further inquiries can be directed to the corresponding author/s.
