# Supplementary material for: Acute caffeine supplementation in rugby players: a systematic review and meta-analysis of physical performance, sport-specific performance, perceptual responses, and physiological markers
Source: Front Nutr. 2026 Jul 3;13:1884686. doi: 10.3389/fnut.2026.1884686 (PMC13375779; doi:10.3389/fnut.2026.1884686)
Supplement: Supplementary file 1 [file Supplementary_file_1.docx]

***Supplementary Material***

**Supplementary Table 1.** PRISMA Checklist.

| **Section and Topic** | **Item #** | **Checklist item** | **Location where item is reported** |
| --- | --- | --- | --- |
| **Acute Caffeine Supplementation in Rugby Players: A Systematic Review and Meta-Analysis of Physical Performance, Sport-Specific Performance and Physiological Responses** | | | 1 |
| Title | 1 | Identify the report as a systematic review. | 1 |
| **ABSTRACT** | | |  |
| Abstract | 2 | See the PRISMA 2020 for Abstracts checklist. | 1, 2 |
| **INTRODUCTION** | | |  |
| Rationale | 3 | Describe the rationale for the review in the context of existing knowledge. | 2,3 |
| Objectives | 4 | Provide an explicit statement of the objective(s) or question(s) the review addresses. | 3 |
| **METHODS** | | |  |
| Eligibility criteria | 5 | Specify the inclusion and exclusion criteria for the review and how studies were grouped for the syntheses. | 3 |
| Information sources | 6 | Specify all databases, registers, websites, organisations, reference lists and other sources searched or consulted to identify studies. Specify the date when each source was last searched or consulted. | 3 |
| Search strategy | 7 | Present the full search strategies for all databases, registers and websites, including any filters and limits used. | Supplementary Table 2 |
| Selection process | 8 | Specify the methods used to decide whether a study met the inclusion criteria of the review, including how many reviewers screened each record and each report retrieved, whether they worked independently, and if applicable, details of automation tools used in the process. | 3 |
| Data collection process | 9 | Specify the methods used to collect data from reports, including how many reviewers collected data from each report, whether they worked independently, any processes for obtaining or confirming data from study investigators, and if applicable, details of automation tools used in the process. | 3 |
| Data items | 10a | List and define all outcomes for which data were sought. Specify whether all results that were compatible with each outcome domain in each study were sought (e.g. for all measures, time points, analyses), and if not, the methods used to decide which results to collect. | 3, 4 |
|  | 10b | List and define all other variables for which data were sought (e.g. participant and intervention characteristics, funding sources). Describe any assumptions made about any missing or unclear information. | 3, 4 |
| Study risk of bias assessment | 11 | Specify the methods used to assess risk of bias in the included studies, including details of the tool(s) used, how many reviewers assessed each study and whether they worked independently, and if applicable, details of automation tools used in the process. | 4 |
| Effect measures | 12 | Specify for each outcome the effect measure(s) (e.g. risk ratio, mean difference) used in the synthesis or presentation of results. | 4 |
| Synthesis methods | 13a | Describe the processes used to decide which studies were eligible for each synthesis (e.g. tabulating the study intervention characteristics and comparing against the planned groups for each synthesis (item #5)). | 3, 4 |
|  | 13b | Describe any methods required to prepare the data for presentation or synthesis, such as handling of missing summary statistics, or data conversions. | 3, 4 |
|  | 13c | Describe any methods used to tabulate or visually display results of individual studies and syntheses. | 4, 5 |
|  | 13d | Describe any methods used to synthesize results and provide a rationale for the choice(s). If meta-analysis was performed, describe the model(s), method(s) to identify the presence and extent of statistical heterogeneity, and software package(s) used. | 4, 5 |
|  | 13e | Describe any methods used to explore possible causes of heterogeneity among study results (e.g. subgroup analysis, meta-regression). | 4, 10-14 |
|  | 13f | Describe any sensitivity analyses conducted to assess robustness of the synthesized results. | Supplementary Figures 15-21 |
| Reporting bias assessment | 14 | Describe any methods used to assess risk of bias due to missing results in a synthesis (arising from reporting biases). | 4, 5, 11, 14 |
| Certainty assessment | 15 | Describe any methods used to assess certainty (or confidence) in the body of evidence for an outcome. | 4, 12, 15 |
| **RESULTS** | | |  |
| Study selection | 16a | Describe the results of the search and selection process, from the number of records identified in the search to the number of studies included in the review, ideally using a flow diagram. | 5 |
|  | 16b | Cite studies that might appear to meet the inclusion criteria, but which were excluded, and explain why they were excluded. | 5 |
| Study characteristics | 17 | Cite each included study and present its characteristics. | 5-9 |
| Risk of bias in studies | 18 | Present assessments of risk of bias for each included study. | 5, 10 |
| Results of individual studies | 19 | For all outcomes, present, for each study: (a) summary statistics for each group (where appropriate) and (b) an effect estimate and its precision (e.g. confidence/credible interval), ideally using structured tables or plots. | 6-14 |
| Results of syntheses | 20a | For each synthesis, briefly summarise the characteristics and risk of bias among contributing studies. | 5-10 |
|  | 20b | Present results of all statistical syntheses conducted. If meta-analysis was done, present for each the summary estimate and its precision (e.g. confidence/credible interval) and measures of statistical heterogeneity. If comparing groups, describe the direction of the effect. | 9-11 |
|  | 20c | Present results of all investigations of possible causes of heterogeneity among study results. | 10-14 |
|  | 20d | Present results of all sensitivity analyses conducted to assess the robustness of the synthesized results. | 11, 12 |
| Reporting biases | 21 | Present assessments of risk of bias due to missing results (arising from reporting biases) for each synthesis assessed. | 11, 14 |
| Certainty of evidence | 22 | Present assessments of certainty (or confidence) in the body of evidence for each outcome assessed. | 12, 15 |
| **DISCUSSION** | | |  |
| Discussion | 23a | Provide a general interpretation of the results in the context of other evidence. | 13-17 |
|  | 23b | Discuss any limitations of the evidence included in the review. | 17, 18 |
|  | 23c | Discuss any limitations of the review processes used. | 17, 18 |
|  | 23d | Discuss implications of the results for practice, policy, and future research. | 17, 18 |
| **OTHER INFORMATION** | | |  |
| Registration and protocol | 24a | Provide registration information for the review, including register name and registration number, or state that the review was not registered. | 2, 3 |
|  | 24b | Indicate where the review protocol can be accessed, or state that a protocol was not prepared. | 2, 3 |
|  | 24c | Describe and explain any amendments to information provided at registration or in the protocol. | n/a |
| Support | 25 | Describe sources of financial or non-financial support for the review, and the role of the funders or sponsors in the review. | 18 |
| Competing interests | 26 | Declare any competing interests of review authors. | 18 |
| Availability of data, code and other materials | 27 | Report which of the following are publicly available and where they can be found: template data collection forms; data extracted from included studies; data used for all analyses; analytic code; any other materials used in the review. | 18 |

**Supplementary Table 2.** Search Strategy.

| **Database** | **Specificities of the database** | **Search strategy** |
| --- | --- | --- |
| PubMed | MeSH terms and Title/Abstract | ("Rugby"[Mesh] OR rugby[Title/Abstract] OR "rugby union"[Title/Abstract] OR "rugby league"[Title/Abstract] OR "rugby sevens"[Title/Abstract] OR "rugby football"[Title/Abstract]) AND ("Caffeine"[Mesh] OR caffeine[Title/Abstract] OR caffeinated[Title/Abstract] OR coffee[Title/Abstract] OR "Coffee"[Mesh] OR "Energy Drinks"[Mesh] OR "energy drink"[Title/Abstract] OR "energy drinks"[Title/Abstract] OR "caffeine gum"[Title/Abstract] OR "caffeinated gum"[Title/Abstract] OR "caffeinated chewing gum"[Title/Abstract] OR "mouth rinse"[Title/Abstract] OR "mouth rinsing"[Title/Abstract]) |
| Web of Science Core Collection | Topic | TS=("rugby" OR "rugby union" OR "rugby league" OR "rugby sevens" OR "rugby football") AND TS=("caffeine" OR "caffeinated" OR "coffee" OR "energy drink" OR "energy drinks" OR "caffeine gum" OR "caffeinated gum" OR "caffeinated chewing gum" OR "mouth rinse" OR "mouth rinsing") |
| Cochrane Library | Title, abstract and keywords | (rugby OR "rugby union" OR "rugby league" OR "rugby sevens" OR "rugby football"):ti,ab,kw AND (caffeine OR caffeinat* OR coffee OR "energy drink" OR "energy drinks" OR "caffeine gum" OR "caffeinated gum" OR "caffeinated chewing gum" OR "mouth rinse" OR "mouth rinsing"):ti,ab,kw |
| Scopus | Title, abstract and keywords | TITLE-ABS-KEY(rugby OR "rugby union" OR "rugby league" OR "rugby sevens" OR "rugby football") AND TITLE-ABS-KEY(caffeine OR caffeinat* OR coffee OR "energy drink*" OR "caffeine gum" OR "caffeinated gum" OR "caffeinated chewing gum" OR "mouth rinse" OR "mouth rinsing") |
| Embase | Emtree terms and title/abstract/author keywords | ('rugby'/exp OR rugby:ti,ab,kw OR 'rugby union':ti,ab,kw OR 'rugby league':ti,ab,kw OR 'rugby sevens':ti,ab,kw OR 'rugby football':ti,ab,kw) AND ('caffeine'/exp OR 'coffee'/exp OR 'energy drink'/exp OR caffeine:ti,ab,kw OR caffeinat*:ti,ab,kw OR coffee:ti,ab,kw OR 'energy drink':ti,ab,kw OR 'energy drinks':ti,ab,kw OR 'caffeine gum':ti,ab,kw OR 'caffeinated gum':ti,ab,kw OR 'caffeinated chewing gum':ti,ab,kw OR 'mouth rinse':ti,ab,kw OR 'mouth rinsing':ti,ab,kw) |

**Supplementary Table 3.** PEDro quality assessment of the included randomized controlled trials.

| **Study** | **First author** | **Year** | **D1** | **D2** | **D3** | **D4** | **D5** | **D6** | **D7** | **D8** | **D9** | **D10** | **D11** | **Total** |
| --- | --- | --- | --- | --- | --- | --- | --- | --- | --- | --- | --- | --- | --- | --- |
| **1** | **Stuart** | **2005** | **1** | **1** | **0** | **1** | **1** | **1** | **0** | **1** | **0** | **1** | **1** | **7** |
| **2** | **Roberts** | **2010** | **1** | **1** | **0** | **1** | **1** | **1** | **0** | **1** | **0** | **1** | **1** | **7** |
| **3** | **Cook** | **2011** | **1** | **1** | **0** | **1** | **1** | **1** | **0** | **1** | **0** | **1** | **1** | **7** |
| **4** | **Del Coso** | **2013** | **1** | **1** | **1** | **1** | **1** | **1** | **1** | **1** | **1** | **1** | **1** | **10** |
| **5** | **Assi** | **2014** | **1** | **1** | **1** | **0** | **1** | **1** | **1** | **0** | **1** | **1** | **1** | **8** |
| **6** | **Portillo** | **2017** | **1** | **1** | **1** | **1** | **1** | **1** | **0** | **1** | **1** | **1** | **1** | **9** |
| **7** | **Wellington** | **2017** | **1** | **1** | **1** | **1** | **1** | **1** | **1** | **0** | **0** | **1** | **1** | **8** |
| **8** | **Clarke** | **2019** | **1** | **1** | **1** | **0** | **1** | **1** | **1** | **1** | **0** | **1** | **1** | **8** |
| **9** | **Ranchordas** | **2019** | **1** | **1** | **1** | **0** | **1** | **1** | **1** | **1** | **0** | **1** | **1** | **8** |
| **10** | **Russell** | **2020** | **1** | **1** | **1** | **1** | **1** | **1** | **1** | **1** | **1** | **1** | **1** | **10** |
| **11** | **Tamilio** | **2022** | **1** | **1** | **1** | **0** | **1** | **1** | **1** | **1** | **0** | **0** | **1** | **7** |
| **12** | **Tallis** | **2024** | **1** | **1** | **1** | **0** | **1** | **1** | **1** | **1** | **0** | **1** | **1** | **8** |
| **13** | **Hsueh** | **2025** | **1** | **1** | **1** | **0** | **1** | **1** | **1** | **1** | **0** | **0** | **1** | **7** |
| **Table note: 1 = criterion satisfied; 0 = criterion not satisfied. D1 is not included in the total PEDro score.**  **D1 = eligibility criteria were specified;**  **D2 = subjects were randomly allocated to groups;**  **D3 = allocation was concealed;**  **D4 = the groups were similar at baseline regarding the most important prognostic indicators;**  **D5 = there was blinding of all subjects; D6 = there was blinding of all therapists or intervention providers;**  **D7 = there was blinding of all assessors who measured at least one key outcome;**  **D8 = measures of at least one key outcome were obtained from more than 85% of the subjects initially allocated to groups;**  **D9 = all subjects for whom outcome measures were available received the treatment or control condition as allocated, or data were analyzed by intention-to-treat;**  **D10 = the results of between-group statistical comparisons were reported for at least one key outcome;**  **D11 = the study provided both point measures and measures of variability for at least one key outcome.**  **D1 is not included in the total PEDro score; the total score is calculated from D2 to D11, with a maximum score of 10.** | | | | | | | | | | | | | | |

**Supplementary Table 4.** JBI quality assessment of the included observational studies.

| **Study** | **The first author** | **Year of Publication** | **JBI-1** | **JBI-2** | **JBI-3** | **JBI-4** | **JBI-5** | **JBI-6** | **JBI-7** | **JBI-8** | **Total** |
| --- | --- | --- | --- | --- | --- | --- | --- | --- | --- | --- | --- |
| **1** | **Dunican** | **2018** | **Y** | **Y** | **Y** | **Y** | **Y** | **N** | **Y** | **Y** | **7/8** |
| **2** | **Caia** | **2022** | **Y** | **Y** | **Y** | **Y** | **Y** | **N** | **Y** | **Y** | **7/8** |
| **Table note:**  **JBI-1 = clearly defined inclusion criteria;**  **JBI-2 = study subjects and setting described in detail;**  **JBI-3 = exposure measured in a valid and reliable way;**  **JBI-4 = objective and standard criteria used for measurement;**  **JBI-5 = confounding factors identified;**  **JBI-6 = strategies used to deal with confounding factors;**  **JBI-7 = outcomes measured in a valid and reliable way;**  **JBI-8 = appropriate statistical analysis.**  **Y = yes; N = no; U = unclear; NA = not applicable.** | | | | | | | | | | | |


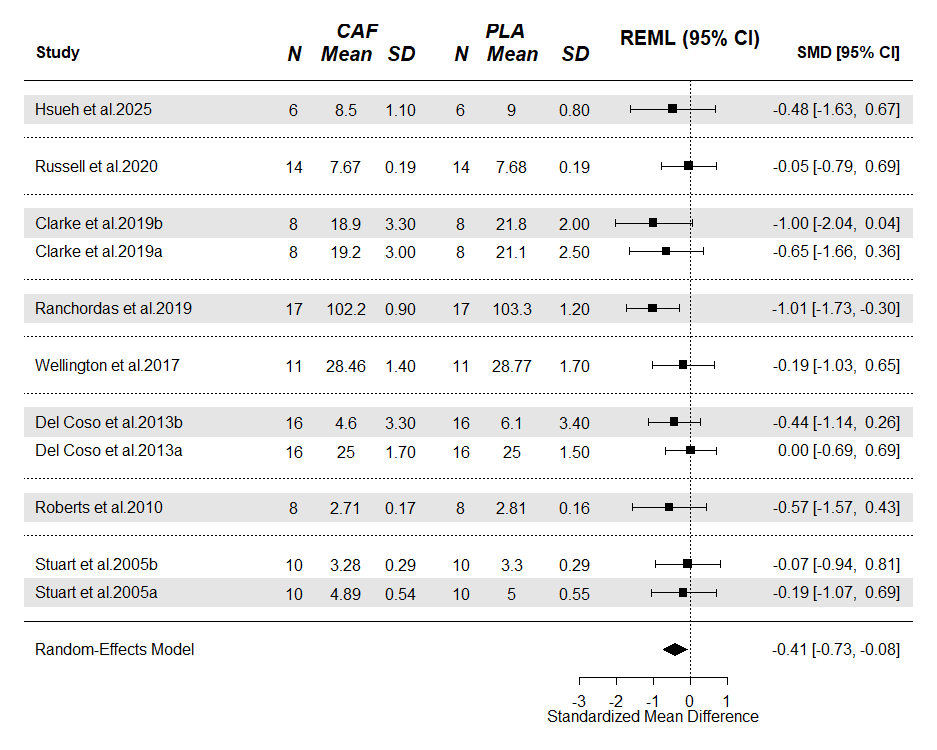


**Supplementary Figure 1.** Forest plot of the effect of acute caffeine supplementation on sprint performance in rugby players. CAF, caffeine condition; PLA, placebo condition; SMD, standardized mean difference; CI, confidence interval. Negative effect sizes indicate improved sprint performance.


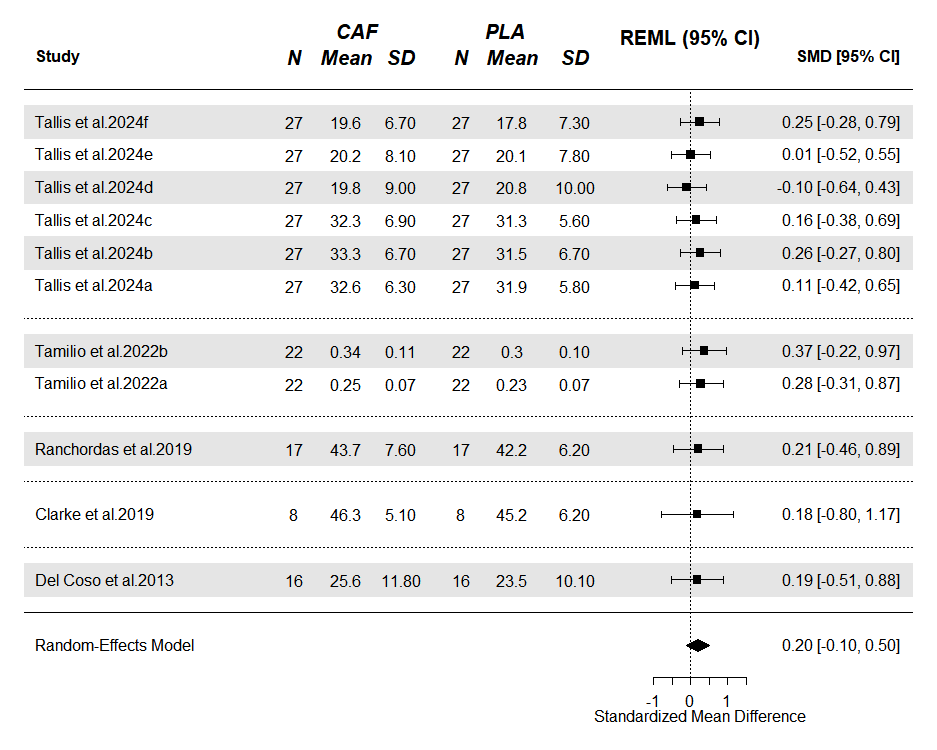


**Supplementary Figure 2.** Forest plot of the effect of acute caffeine supplementation on jumping performance in rugby players. CAF, caffeine condition; PLA, placebo condition; SMD, standardized mean difference; CI, confidence interval. Positive effect sizes indicate improved jumping performance.


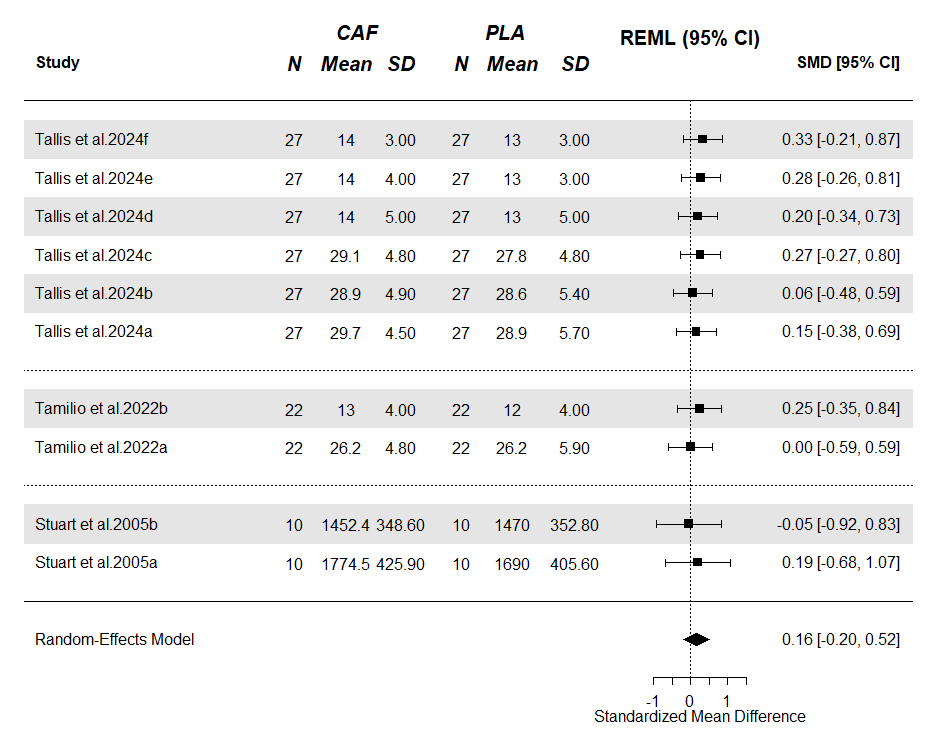


**Supplementary Figure 3.** Forest plot of the effect of acute caffeine supplementation on strength performance in rugby players. CAF, caffeine condition; PLA, placebo condition; SMD, standardized mean difference; CI, confidence interval. Positive effect sizes indicate improved strength performance.
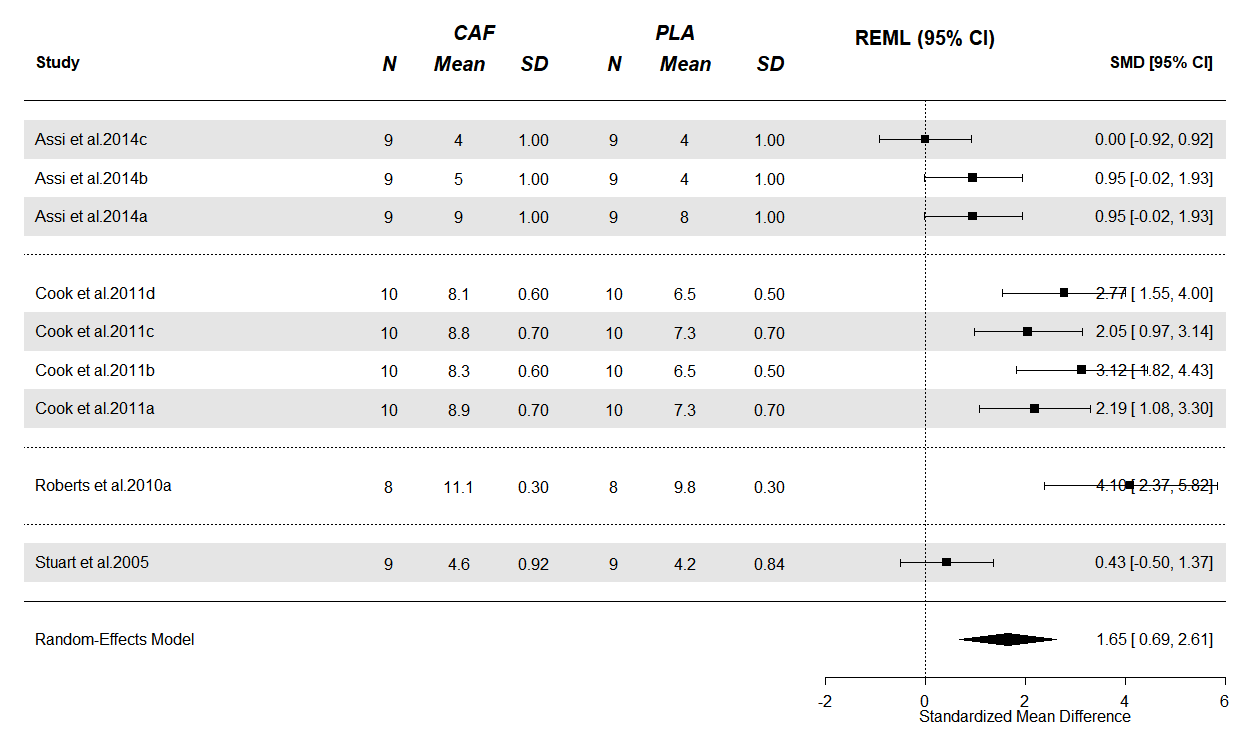


**Supplementary Figure 4.** Forest plot of the effect of acute caffeine supplementation on passing accuracy in rugby players. CAF, caffeine condition; PLA, placebo condition; SMD, standardized mean difference; CI, confidence interval. Positive effect sizes indicate improved passing accuracy.


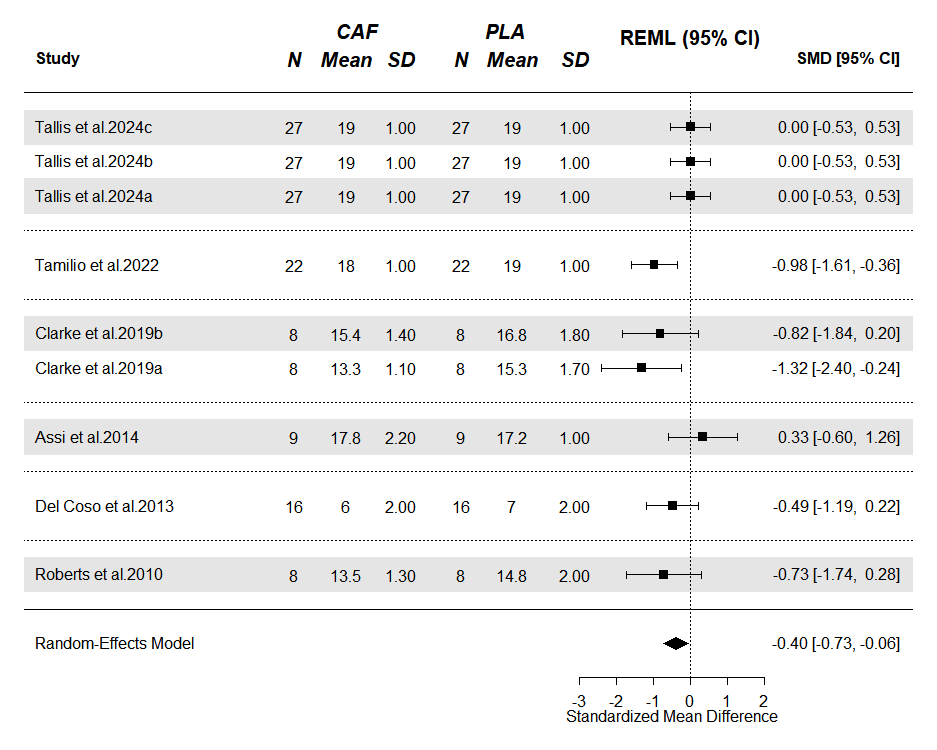


**Supplementary Figure 5.** Forest plot of the effect of acute caffeine supplementation on rating of perceived exertion in rugby players. CAF, caffeine condition; PLA, placebo condition; RPE, rating of perceived exertion; SMD, standardized mean difference; CI, confidence interval. Negative effect sizes indicate lower RPE.


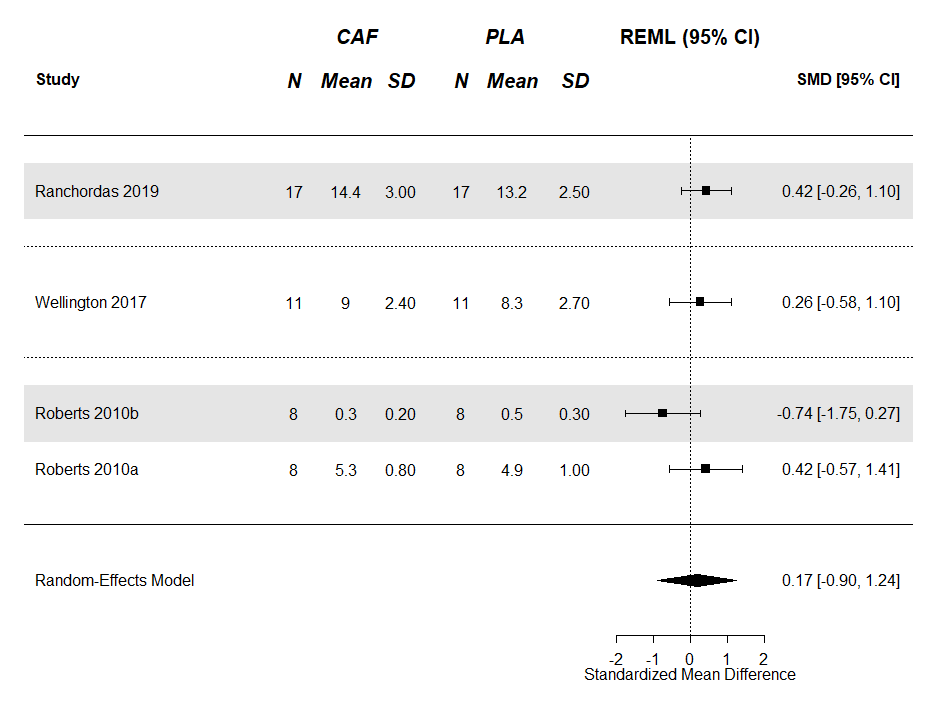


**Supplementary Figure 6.** Forest plot of the effect of acute caffeine supplementation on metabolic markers in rugby players. CAF, caffeine condition; PLA, placebo condition; SMD, standardized mean difference; CI, confidence interval. The direction of effect should be interpreted according to the specific metabolic marker.


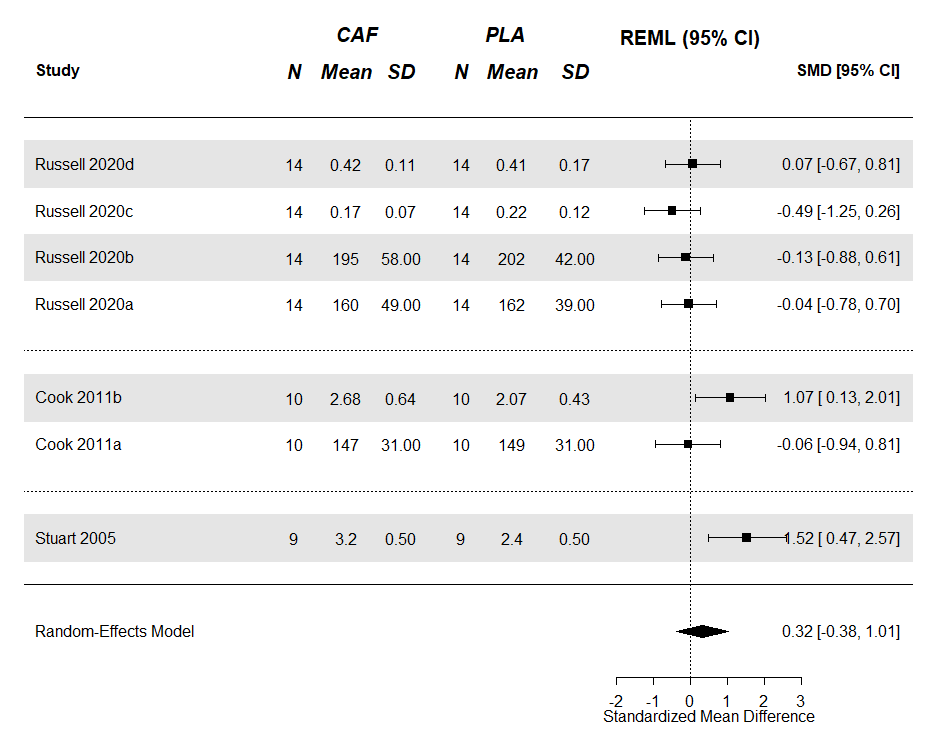


**Supplementary Figure 7.** Forest plot of the effect of acute caffeine supplementation on hormonal markers in rugby players. CAF, caffeine condition; PLA, placebo condition; SMD, standardized mean difference; CI, confidence interval. The direction of effect should be interpreted according to the specific hormonal marker.


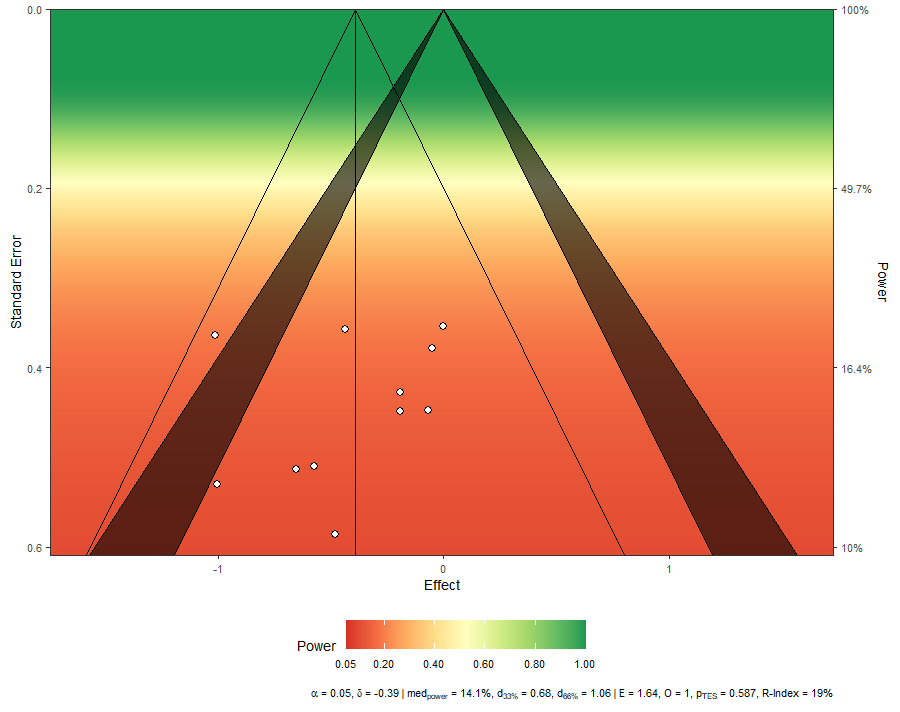


**Supplementary Figure 8.** Power-enhanced funnel plot (sunset plot) for sprint performance. Each point represents an effect size. The background color gradient indicates the approximate statistical power, with warmer colors reflecting lower power and greener colors reflecting higher power. This plot was used as an exploratory visual aid for assessing small-study effects and publication-bias-related patterns in sprint performance.


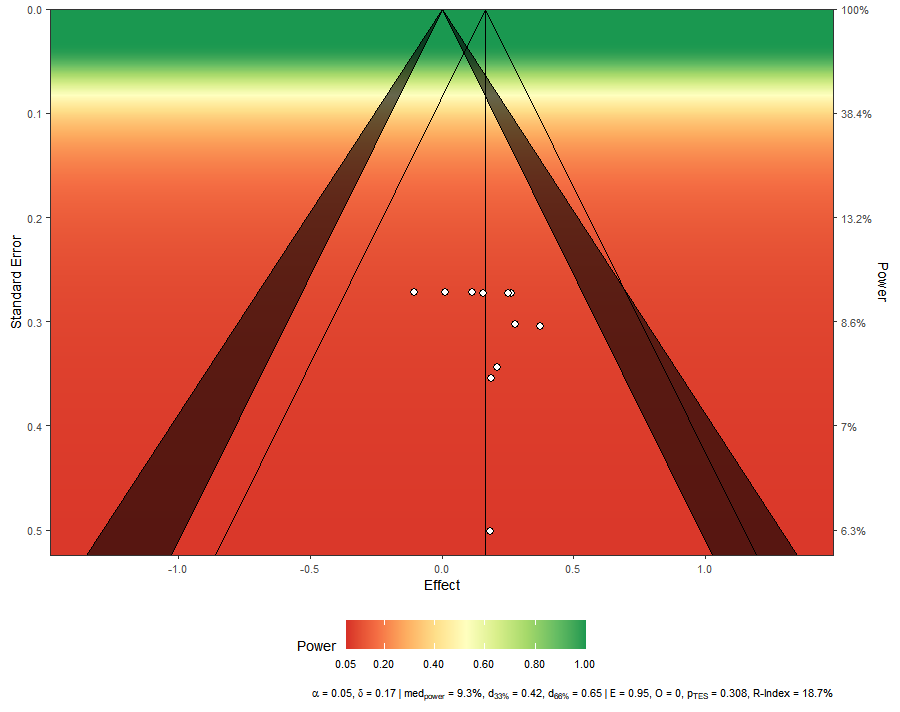


**Supplementary Figure 9.** Power-enhanced funnel plot (sunset plot) for jumping performance. Each point represents an effect size. The background color gradient indicates the approximate statistical power, with warmer colors reflecting lower power and greener colors reflecting higher power. This plot was used as an exploratory visual aid for assessing small-study effects and publication-bias-related patterns in jumping performance.


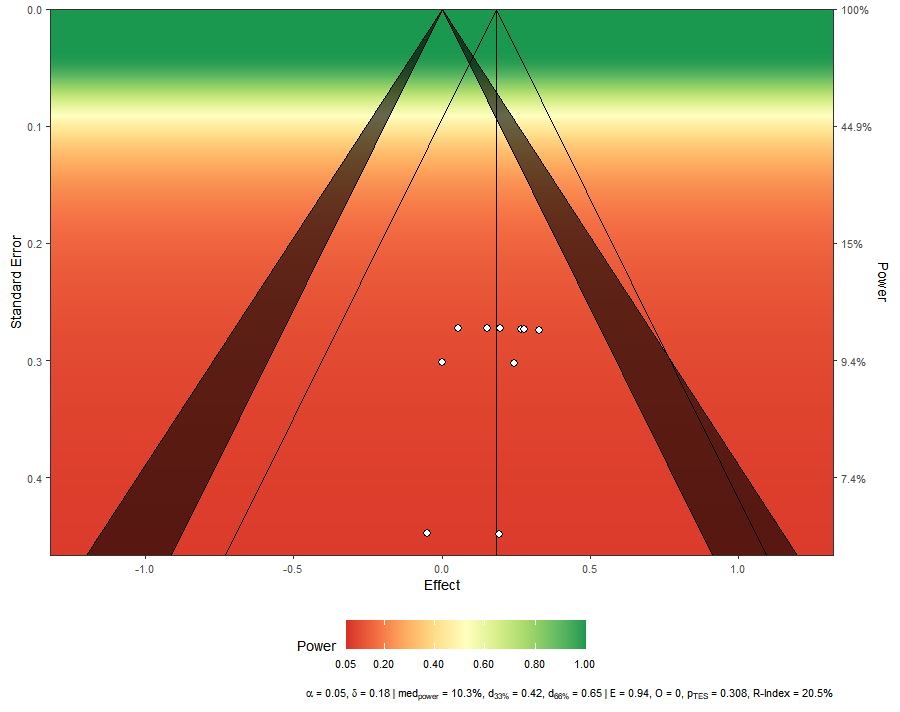


**Supplementary Figure 10.** Power-enhanced funnel plot (sunset plot) for strength performance. Each point represents an effect size. The background color gradient indicates the approximate statistical power, with warmer colors reflecting lower power and greener colors reflecting higher power. This plot was used as an exploratory visual aid for assessing small-study effects and publication-bias-related patterns in strength performance.


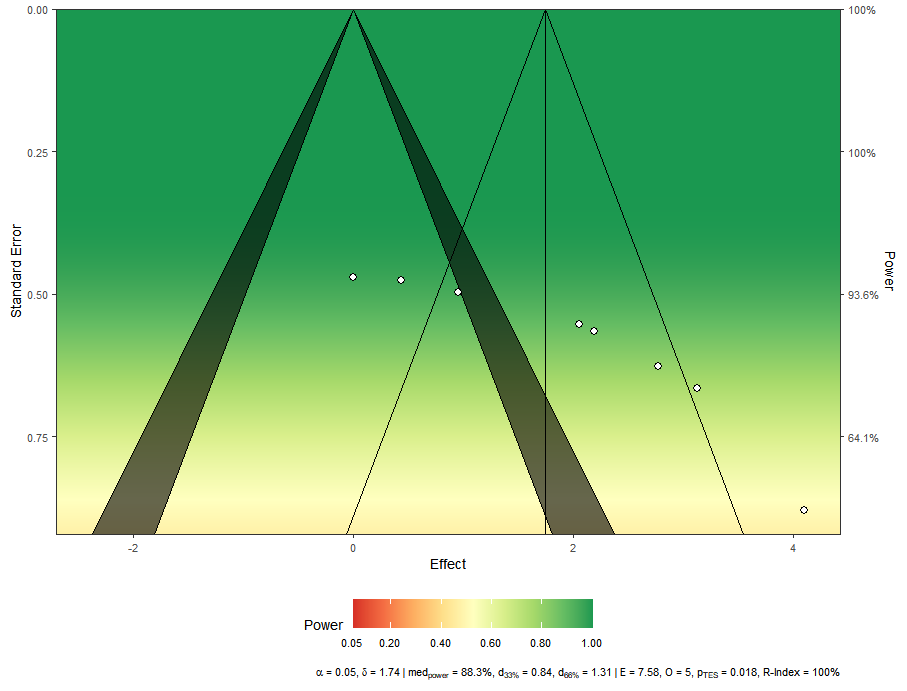


**Supplementary Figure 11.** Power-enhanced funnel plot (sunset plot) for passing accuracy. Each point represents an effect size. The background color gradient indicates the approximate statistical power, with warmer colors reflecting lower power and greener colors reflecting higher power. This plot was used as an exploratory visual aid for assessing small-study effects and publication-bias-related patterns in passing accuracy.


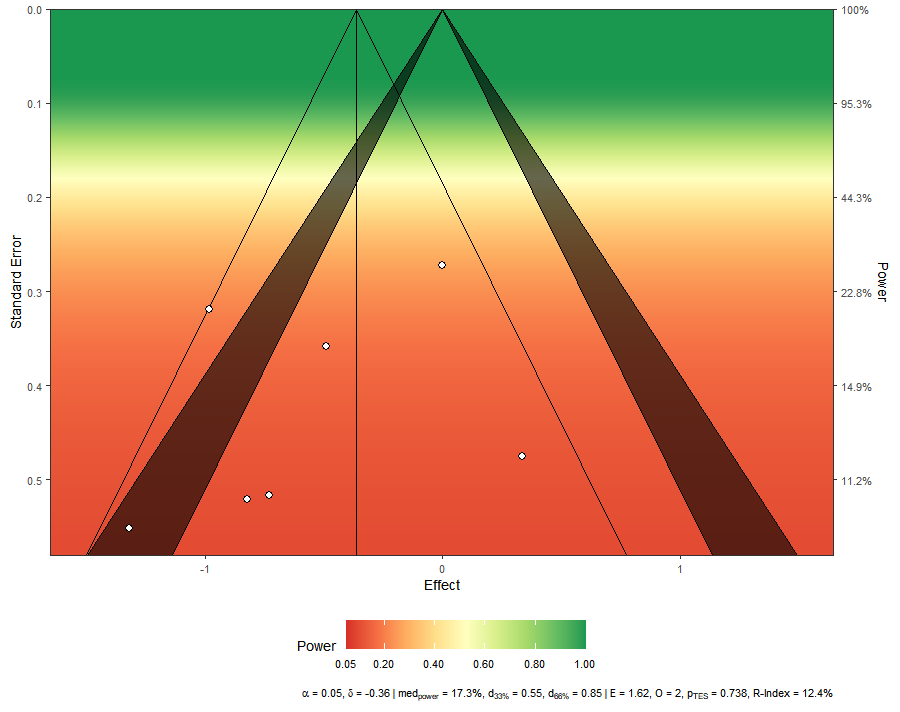


**Supplementary Figure 12.** Power-enhanced funnel plot (sunset plot) for RPE. Each point represents an effect size. The background color gradient indicates the approximate statistical power, with warmer colors reflecting lower power and greener colors reflecting higher power. This plot was used as an exploratory visual aid for assessing small-study effects and publication-bias-related patterns in RPE.


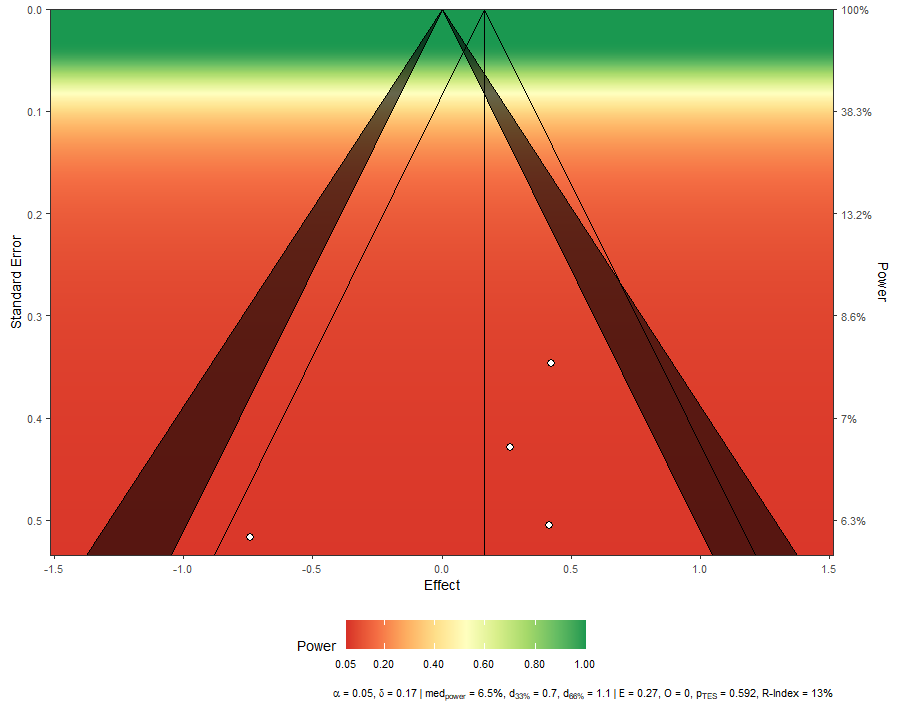


**Supplementary Figure 13.** Power-enhanced funnel plot (sunset plot) for metabolic markers. Each point represents an effect size. The background color gradient indicates the approximate statistical power, with warmer colors reflecting lower power and greener colors reflecting higher power. This plot was used as an exploratory visual aid for assessing small-study effects and publication-bias-related patterns in metabolic markers.


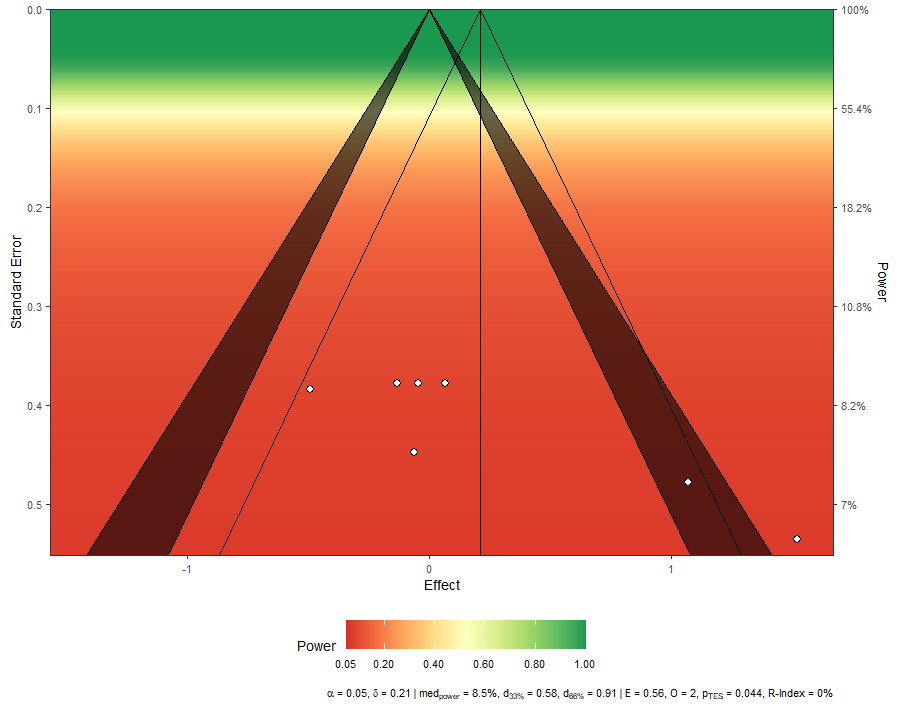


**Supplementary Figure 14.** Power-enhanced funnel plot (sunset plot) for hormonal markers. Each point represents an effect size. The background color gradient indicates the approximate statistical power, with warmer colors reflecting lower power and greener colors reflecting higher power. This plot was used as an exploratory visual aid for assessing small-study effects and publication-bias-related patterns in hormonal markers.


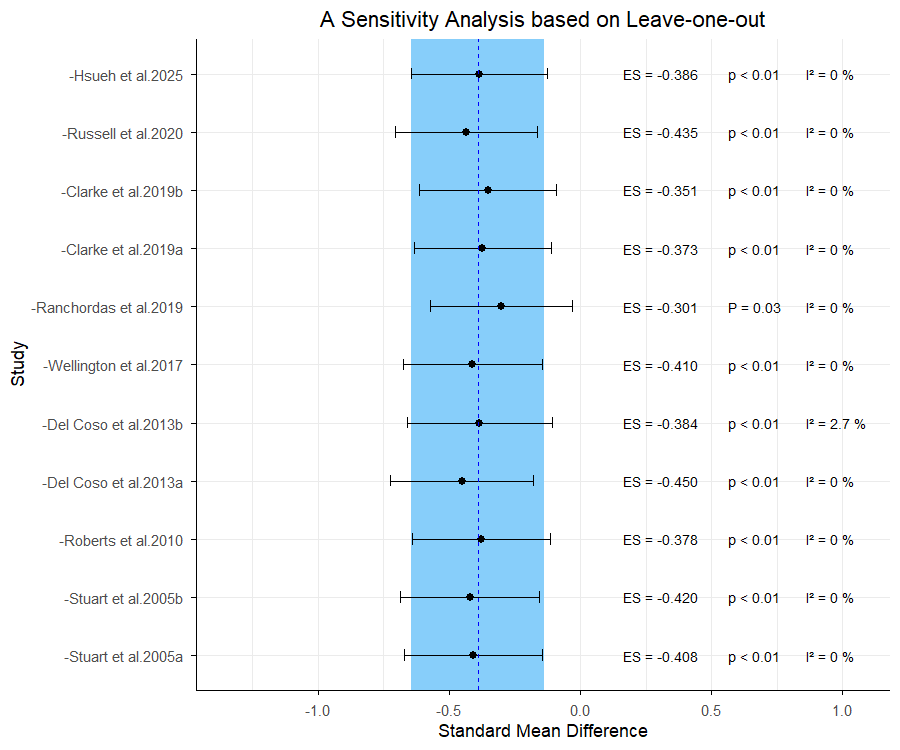


**Supplementary Figure 15.** Leave-one-out sensitivity analysis of sprint performance.


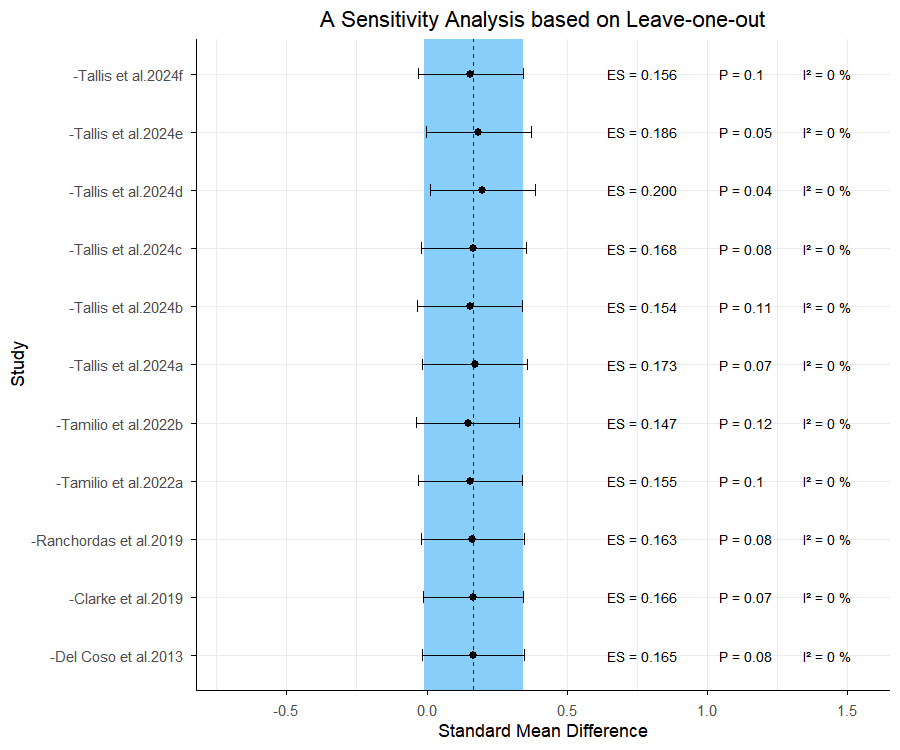


**Supplementary Figure 16.** Leave-one-out sensitivity analysis of jumping performance.


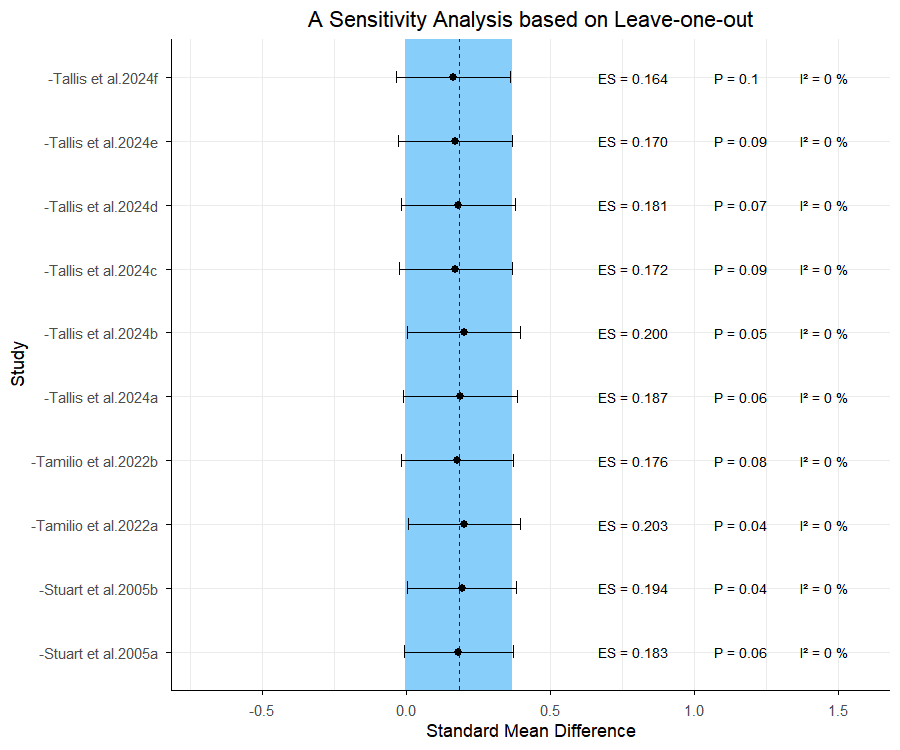


**Supplementary Figure 17.** Leave-one-out sensitivity analysis of strength performance.


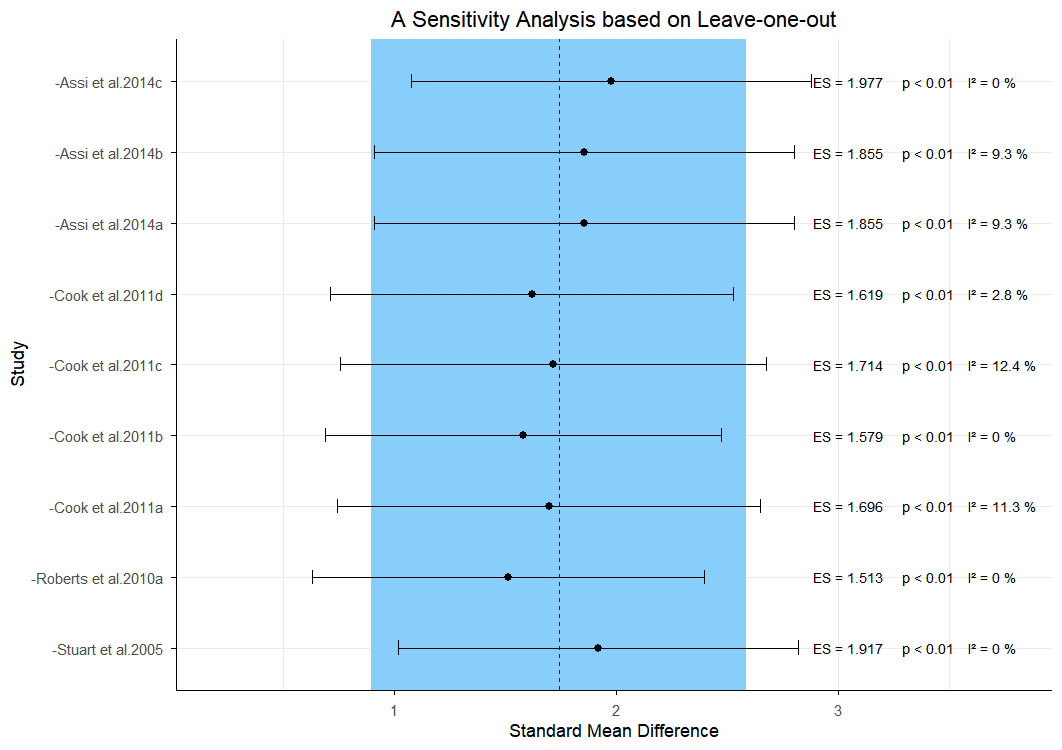


**Supplementary Figure 18.** Leave-one-out sensitivity analysis of passing accuracy.


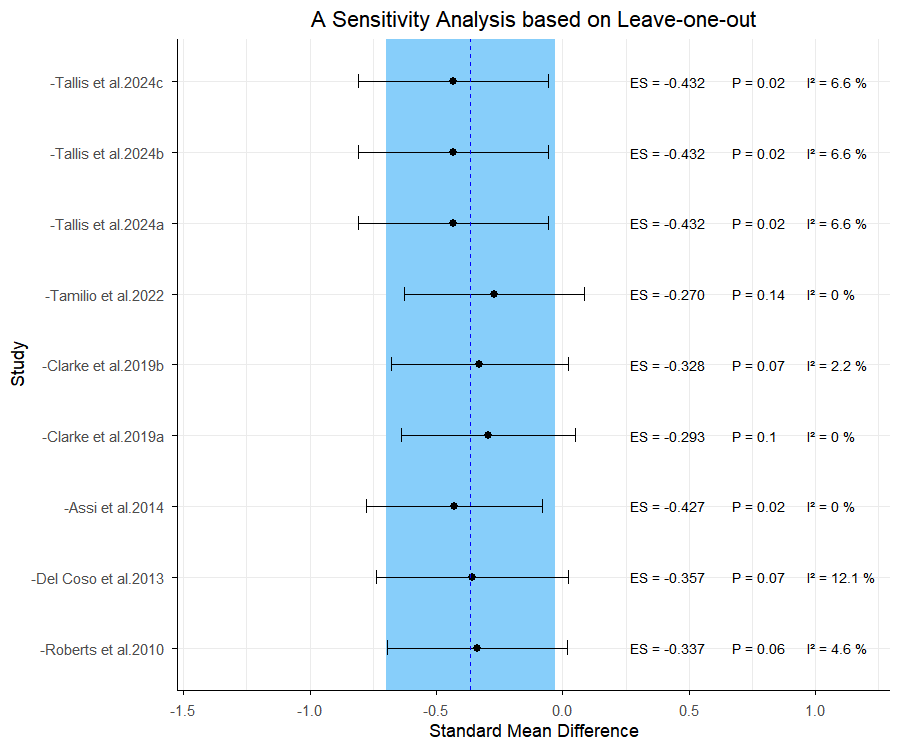


**Supplementary Figure 19.** Leave-one-out sensitivity analysis of RPE.


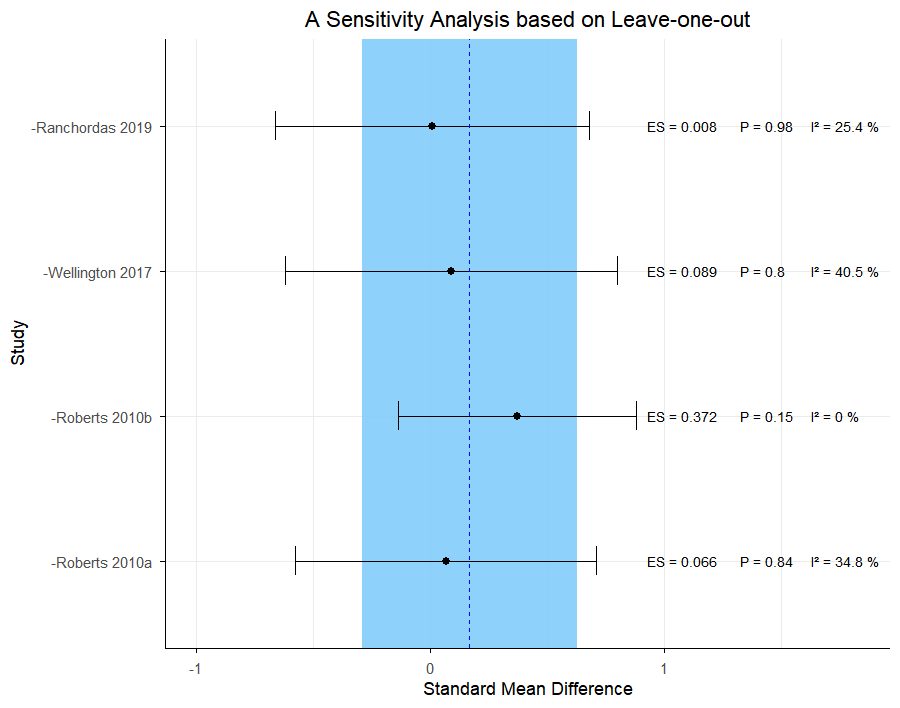


**Supplementary Figure 20.** Leave-one-out sensitivity analysis of metabolic markers.


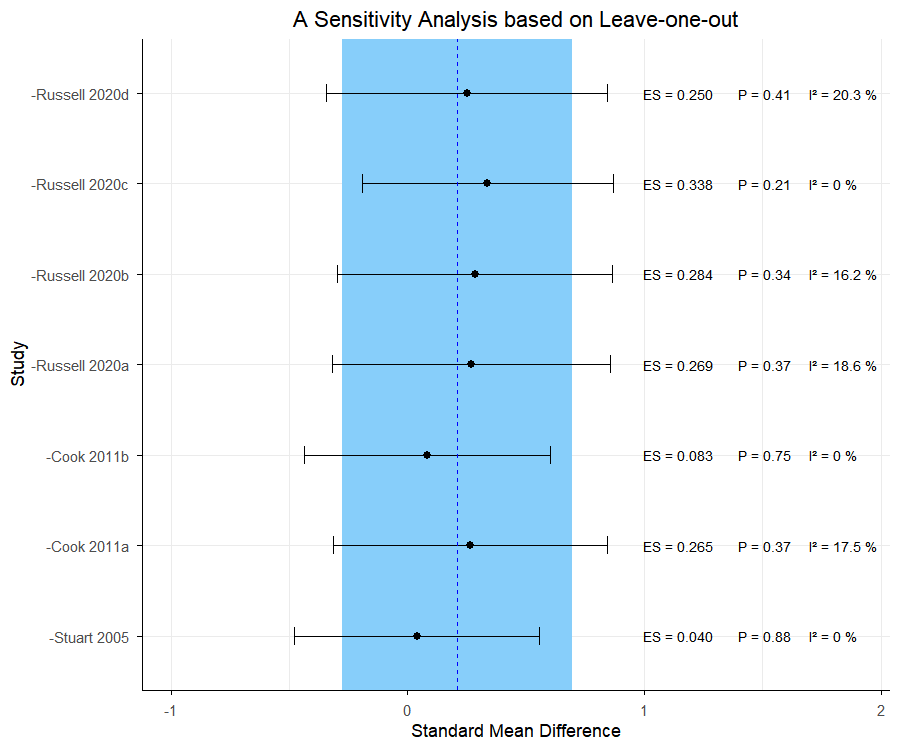


**Supplementary Figure 21.** Leave-one-out sensitivity analysis of hormonal markers.
